# Accelerated polyethylene biodegradation in soil through carbon nanomaterial-primed rhizospheres

**DOI:** 10.1016/j.ese.2026.100772

**Published:** 2026-09-23

**Authors:** Haoran Liu, Lena Ciric, Manpreet Bhatti

**Affiliations:** UCL Department of Civil, Environmental and Geomatic Engineering, London, WC1E 6BT, UK

**Keywords:** Polyethylene degradation, Carbon nanomaterials, Rhizosphere priming, Polymer breakdown, Phytotoxicity alleviation

## Abstract

Polyethylene accounts for the majority of terrestrial plastic waste, yet its chemical inertness resists natural decomposition in soil. Rhizoremediation offers a strategy that combines plant and microbe activity, but recalcitrant C–C backbones create a kinetic bottleneck that historically restricts plastic mass loss to under 3%. Here we show that engineered carbon nanomaterials prime the plant rhizosphere to breach this kinetic barrier, driving accelerated polyethylene biodegradation in soil. Multi-walled carbon nanotubes accelerated surface-layer breakdown, achieving 12.1% mass loss within 90 days, compared to less than 0.1% in abiotic controls and 0.6% in unamended soil. This nanomaterial-amended degradation was accompanied by a 97% reduction in tensile modulus, from 782.8 to 19.6 MPa, and surface oxidation, with the O 1 s/C 1s ratio increasing from 0.17 to 0.49. In deeper soil, graphene oxide sustains long-term degradation (5.8%) tightly synchronized with root penetration. Mechanistically, carbon nano-interfaces recruit pioneer degraders (*Pseudolabrys* and *Rhodanobacter*) and enrich metabolic pathways responsible for C–C bond cleavage (K00059) and intermediate detoxification (K00799). Crucially, the nanomaterials alleviate polyethylene-induced phytotoxicity, enhancing plant biomass and potentially supporting a plant-microbe interaction. These findings demonstrate that abiotic nano-interfaces can prime biological systems to dismantle persistent polymers, offering a scalable blueprint for managing soil plastic contamination.

## Highlights

• Carbon nanomaterials prime the rhizosphere to breach kinetic bottlenecks in soil polyethylene biodegradation.
• MWCNTs drive 12.1% plastic mass loss in 90 days alongside a 97% collapse in polymer tensile modulus.
• Nano-interfaces recruit pioneer degraders and upregulate enzymatic pathways for C–C bond cleavage.
• Nanomaterials alleviate plastic phytotoxicity, sustaining a plant-microbe loop for polymer remediation.

## Graphical abstract

**Figure:**
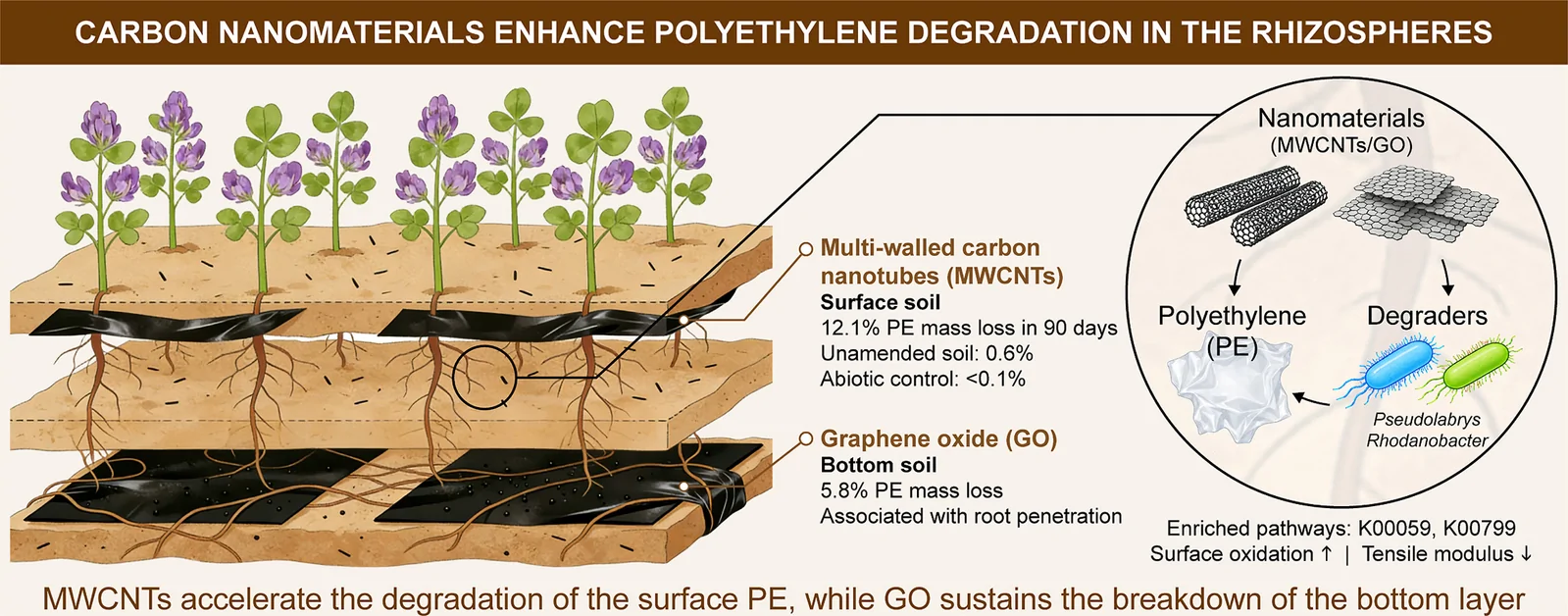

## 1 Introduction

Polyethylene (PE) accounts for approximately 60% of global plastic waste and is consistently the most frequently detected polymer in contaminated terrestrial environments worldwide [1,2], making it a significant socio-ecological problem [3]. Its chemical inertness and resistance to biodegradation lead to long-term accumulation in soils. This persistence has well-documented effects on soil health, biota, and crop productivity [4], particularly in relation to its nanoplastic fractions [5]. Conventional remediation approaches, such as incineration and landfilling, are energy-intensive and may generate secondary pollution, underscoring the need for sustainable, low-impact strategies that integrate with natural ecosystems [5,6]. However, even biodegradable mulch films, which are often promoted as eco-friendly alternatives, involve trade-offs between environmental benefits and production burdens [7].

Phytoremediation and rhizoremediation harness plants and their associated microorganisms to degrade or stabilize pollutants, offering promising alternatives due to their low cost and ecological compatibility [8,9]. Leguminous plants, particularly soybean (*Glycine max* L.) and alfalfa (*Medicago sativa* L.), have shown effective degradation of organic pollutants, including plastic [10,11]. However, quantitative degradation data remain scarce for plant-based plastic remediation, and few available studies have reported relatively low degradation rates. In a wheat-soil system, the maximum PE weight loss was only 2.8% after 100 days, whereas in the soybean rhizosphere, approximately 8% degradation of large poly(butylene adipate-co-terephthalate) (PBAT) microplastics was observed over 70 days [11,12]. These findings underscore the recalcitrance of PE as a major bottleneck for rhizoremediation, highlighting the need for auxiliary strategies.

Carbon-based nanomaterials (NMs), such as multi-walled carbon nanotubes (MWCNTs) and graphene oxide (GO), have attracted considerable attention for their ability to accelerate the degradation of organic pollutants through their high specific surface areas and surface-mediated electron transfer [[13], [14], [15]]. Their catalyst-like acceleration has been well documented in controlled laboratory settings. However, most studies have used short time scales and simplified aqueous or solid-film systems [16,17]. Whether these effects translate into enhanced degradation in complex soil–plant systems remains largely unknown [18]. Furthermore, the vertical heterogeneity of plastic contamination in soil has been largely overlooked. Surface and subsurface films experience different environmental conditions and are influenced differently by root activity, yet few studies have explicitly compared degradation dynamics across soil depths [19,20]. Therefore, the performance of carbon-based NMs over time and across soil depths requires systematic evaluation for rhizoremediation applications.

Here we conducted a 90-day dynamic bioremediation experiment using alfalfa amended with MWCNTs and GO. We compared their effects on PE degradation over time and at different soil depths, evaluated plant growth responses under NM amendment and PE stress, and identified microbial taxa and metabolic pathways associated with enhanced degradation. By integrating these observations, we aimed to clarify how commercial carbon-based NMs can be applied for sustainable plastic remediation while also informing their safe and effective use in soil–plant systems.

## 2 Materials and Methods

### 2.1 Materials

MWCNTs (outer diameter, 5–20 nm; length, 10 μm) and GO (thickness, 0.8–2 nm; lateral size, 5–10 μm) were obtained from Shilpent, India (imported and sale via Desertcart, United Kingdom) and used as model NMs. The detailed physicochemical properties of both NMs are presented in Supplementary Table S1.

Alfalfa seeds were purchased from Amazon, United Kingdom and germinated in soil (Verve Bulb Planting Compost, B&Q, United Kingdom). The soil is a standard peat-free multipurpose compost containing composted green waste, wood fiber, and sand. Supplier-reported properties included a pH of 6.0–7.5, electrical conductivity of 0.5–1.5 mS cm^−1^, organic matter content of 50–85%, and total nitrogen (N), phosphorus (P), and potassium (K) contents of ≥2% each. This soil is widely used for gardening and horticulture in the United Kingdom and is representative of many urban and peri-urban soils.

Commercial black, double-layer linear low-density polyethylene film (Ramofy Ltd, United Kingdom) was purchased from Amazon. The film had a thickness of 20 μm and a density of 0.91–0.93 g cm^−3^ and contained carbon black pigment. This type of film is commonly used for packaging.

### 2.2 Characterization of NMs

The physicochemical properties of the two commercial NMs were characterized before the experiments. We used scanning electron microscopy and atomic force microscopy to examine surface morphology and employed Brunauer–Emmett–Teller analysis to determine specific surface area. Surface functional groups were identified by Fourier-transform infrared spectroscopy (FT-IR), and elemental composition was determined by X-ray photoelectron spectroscopy (XPS). We measured zeta potential to evaluate the colloidal stability and surface charge of the NMs in aqueous solution. Detailed characterization of images and instrument specifications are provided in Supplementary Text, Section I.

### 2.3 Germination test

We conducted a 45-day preliminary hydroponic experiment to determine the appropriate concentration range and assess the potential to promote PE degradation (Fig. 1a and Supplementary Fig. S2a). Based on these results, a germination test was performed to assess the effects of PE, NMs, and their combined exposure on alfalfa seeds (Fig. 1b). Alfalfa seeds were surface-sterilized with 10% hydrogen peroxide for 10 min, rinsed thoroughly with Milli-Q water, and placed on filter paper moistened with NM suspensions at concentrations of 100, 150, and 200 mg L^−1^ in Petri dishes. This concentration range was selected based on preliminary hydroponic experiments at 100 mg L^−1^, which confirmed the feasibility of NM-assisted bioremediation. The higher concentrations (150 and 200 mg L^−1^) were included to assess dose-dependent effects on both degradation efficiency and potential phytotoxicity. Additional treatment groups received PE film (0.1 ± 0.005 g) together with the corresponding NM suspensions to evaluate their combined effects. Control groups received Milli-Q water only. Each treatment comprised three replicate dishes, each containing 20 seeds. Petri dishes were incubated in the dark at 25 °C. Germination potential (GP) and germination rate (GR) were recorded on days 3 and 7, respectively. Seeds were considered germinated when the radicle length exceeded 2 mm.

**Fig. 1:**
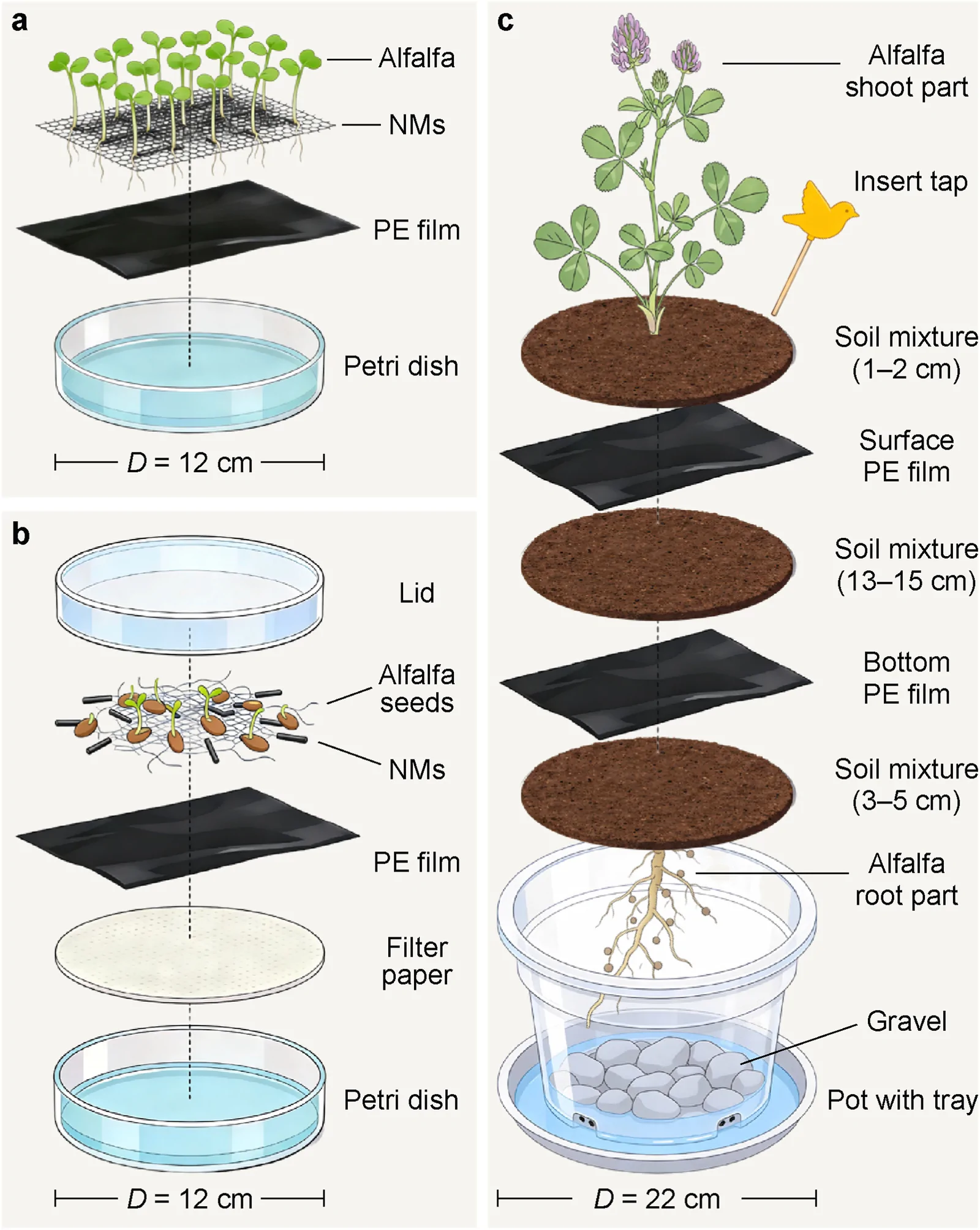
**Experimental designs used to evaluate nanomaterial-assisted polyethylene degradation. a**, Preliminary hydroponic experiment in which alfalfa was exposed to nanomaterials in the presence of a polyethylene (PE) film. **b**, Germination assay conducted in a Petri dish containing alfalfa seeds, nanomaterials, and a PE film. **c**, Pot experiment showing the positions of PE films in the surface and bottom soil layers and the arrangement of the alfalfa shoots, roots, and gravel layer. Photographs of the experimental setups are provided in Supplementary Fig. S2. NMs, nanomaterials; PE, polyethylene.

### 2.4 Plant cultivation and soil preparation

The soil was sieved through a 2 mm mesh to remove debris and homogenized with NMs before use. The concentration gradient of NMs (100, 150, and 200 mg kg^−1^ soil) was determined through sequential batch experiments. Based on the results of our previous review study [2] and the hydroponic screening (Fig. 1a and b), two concentrations (100 and 200 mg kg^−1^) were tested in the first full 90-day batch for both MWCNTs and GO. After observing dose-dependent trends, an intermediate concentration (150 mg kg^−1^) was added in a second independent batch to capture the potential optimal response. All treatments were replicated three times, and the final dataset integrates results from both batches. Three controls were included: PE with plants but without nanomaterials (PE + plant), PE without plants but with nanomaterials (PE + NM) and PE without plants or nanomaterials (PE only).

Each pot (22 cm in diameter) contained 700 ± 10 g of the soil–NM mixture (Fig. 1c). A gravel layer was placed at the bottom of each pot to facilitate drainage and prevent soil loss. Two pieces of PE film (0.7 ± 0.01 g each, approximately 20 cm × 20 cm) were buried in each pot, with one placed at 2 cm below the soil surface and the other 2 cm above the gravel layer, ensuring full coverage of the pot cross-section. Alfalfa seeds were sown on the soil surface and thinned to 25–30 plants per pot after germination (Supplementary Fig. S2c–d). Plants were maintained in a greenhouse at 25 ± 1 °C under a 16 h light/8 h dark photoperiod using growth lights. Pots were watered regularly to maintain consistent soil moisture, and soil pH and moisture content were monitored every 2 days. Insect traps were installed to monitor potential pest interference and were replaced every 30 days. This setup was designed to mimic a high-exposure scenario typical of urban and peri-urban soils contaminated by packaging plastic waste rather than conventional agricultural mulch-film pollution. It reflects typical pollution conditions in the United Kingdom and European cities and urban–rural areas based on our previous study [2].

### 2.5 Sampling and degradation rate measurement

Destructive sampling was performed at 30, 60, and 90 days after planting. At each time point, aboveground plant tissues were harvested, and roots were carefully extracted from the soil. Plant height and root length were measured with a ruler to two decimal places. The tallest and shortest individuals in each pot were excluded, and the mean was calculated from the next three tallest plants. Fresh biomass was determined by weighing all plants from each pot after rinsing with Milli-Q water and removing surface moisture with absorbent paper. The average value was calculated for the three replicate pots. The number of flying insects was also recorded as an additional indicator of greenhouse soil and plant health (Supplementary Fig. S2c). Pots were separated by treatment groups with partitions, which prevented strict randomization of insect trapping. Nevertheless, averaging the counts from adjacent traps yielded data with qualitative value and semi-quantitative comparability across treatments.

Soil samples were collected separately from the surface layer (0–5 cm depth) and bottom layer (17–22 cm depth, below the lower PE film) and immediately frozen at −80 °C for subsequent deoxyribonucleic acid (DNA) extraction. The recovered PE films were rinsed sequentially with copious amounts of Milli-Q water to remove adhering soil particles, dried in a fume hood for 48 h, and weighed. This rinse–dry–weigh cycle was repeated until consecutive measurements showed no significant change in mass, defined as less than 0.1 mg. The recovery rate of PE film without NMs or plants in the soil was 100 ± 0.01% at 90 days. The degradation rate was calculated as the percentage of mass loss relative to the initial film weight [12]. The reliability of this mass-loss approach was supported by the statistically significant differences observed between treatment groups and controls throughout the experiment. Tensile tests were performed on the recovered PE films using a tensile tester based on the BS EN ISO 527-1:2019 standard to determine the elastic modulus (Young's modulus) as an indicator of mechanical stiffness loss after degradation. The recovered PE films were analyzed by FT-IR and XPS to determine surface chemical changes, including oxidation and changes in functional groups [5,21,22]. Details are provided in Supplementary Text, Section I.

### 2.6 Soil microbial community analysis

DNA was extracted from 0.5 g soil samples using the FastDNA Spin Kit for Soil (MP Biomedicals) according to the manufacturer's protocol. DNA concentration and purity were measured with a NanoDrop ND-1000 ultraviolet–visible spectroscopy (UV–Vis) spectrophotometer. The V4–V5 hypervariable region of the bacterial 16S rRNA gene was amplified using primers 515F and 907R. Polymerase chain reaction (PCR) amplification, library preparation, and sequencing were conducted by Novogene (Cambridge, United Kingdom) using the Illumina MiSeq PE250 platform. Raw sequencing reads were deposited in the National Center for Biotechnology Information Sequence Read Archive under accession number PRJNA1378808.

Raw sequencing data were processed using the QIIME pipeline following established quality-control procedures. Quality filtering was performed using fastp with a sliding-window approach, and primer sequences were removed with Cutadapt. Operational taxonomic units were clustered at 97% sequence similarity using the UPARSE algorithm. The operational taxonomic unit table was rarefied to 25,487 sequences per sample, and taxa with a relative abundance below 0.1% were grouped as “Others.” Alpha and beta diversity, heatmaps, co-occurrence networks, and functional gene predictions were used to characterize microbial community structure and metabolic potential.

### 2.7 Statistical analysis

All experiments were conducted with three replicates per treatment at the Environmental Engineering Laboratory, Department of Civil, Environmental and Geomatic Engineering, University College London, United Kingdom. Data were organized in Microsoft Excel and visualized using OriginPro 2021 and R (version 4.5.1). Results are expressed as means ± standard deviations. One-way analysis of variance (ANOVA) followed by Tukey's post-hoc test was used to compare differences among treatments, and two-way ANOVA was used to evaluate the effects of each pair of factors (time × depth, time × NM type, and depth × NM type) on degradation rates and plant growth indicators. Statistical significance was set at *p* < 0.05. All original data used to generate the figures are provided in Supplementary Data.

## 3 Results

### 3.1 Nanomaterial characterization

The physicochemical properties of MWCNTs and GO are summarized in Supplementary Table S1 and Supplementary Fig. S1. Brunauer–Emmett–Teller analysis showed specific surface areas of 89.8 ± 3.2 m^2^ g^−1^ for MWCNTs and 63.6 ± 4.5 m^2^ g^−1^ for GO. Scanning electron microscopy revealed the tubular structure of MWCNTs and the lamellar morphology of GO. Based on the data provided in the manual, both MWCNTs and GO may exhibit agglomeration. Atomic force microscopy further confirmed distinct surface features, with triangular pyramidal protrusions on MWCNTs and a smooth surface on GO. FT-IR spectra showed oxygen (O)-containing functional groups in both NMs, while XPS identified carbon and oxygen as their primary elemental components. These observations are consistent with previous reports [23,24].

### 3.2 Plant growth indicators under plastic stress

The GP and final GR of alfalfa varied among treatments (Fig. 2a). Across all concentrations, PE alone significantly inhibited germination, reducing GP by 32–35% and GR by 16–18% compared with the control (seeds with MQ water only) (*p* < 0.05). In contrast, application of MWCNTs or GO alone had no adverse effects. When co-applied with PE, both NMs alleviated the inhibitory effect. At 100 mg kg^−1^, GP increased from 52.7% under PE exposure alone to approximately 79.5% with either MWCNTs or GO, while GR increased by 7–12 percentage points across concentrations. At 200 mg kg^−1^, GR under both combined treatments was comparable to that of the control, indicating concentration-dependent mitigation.

**Fig. 2:**
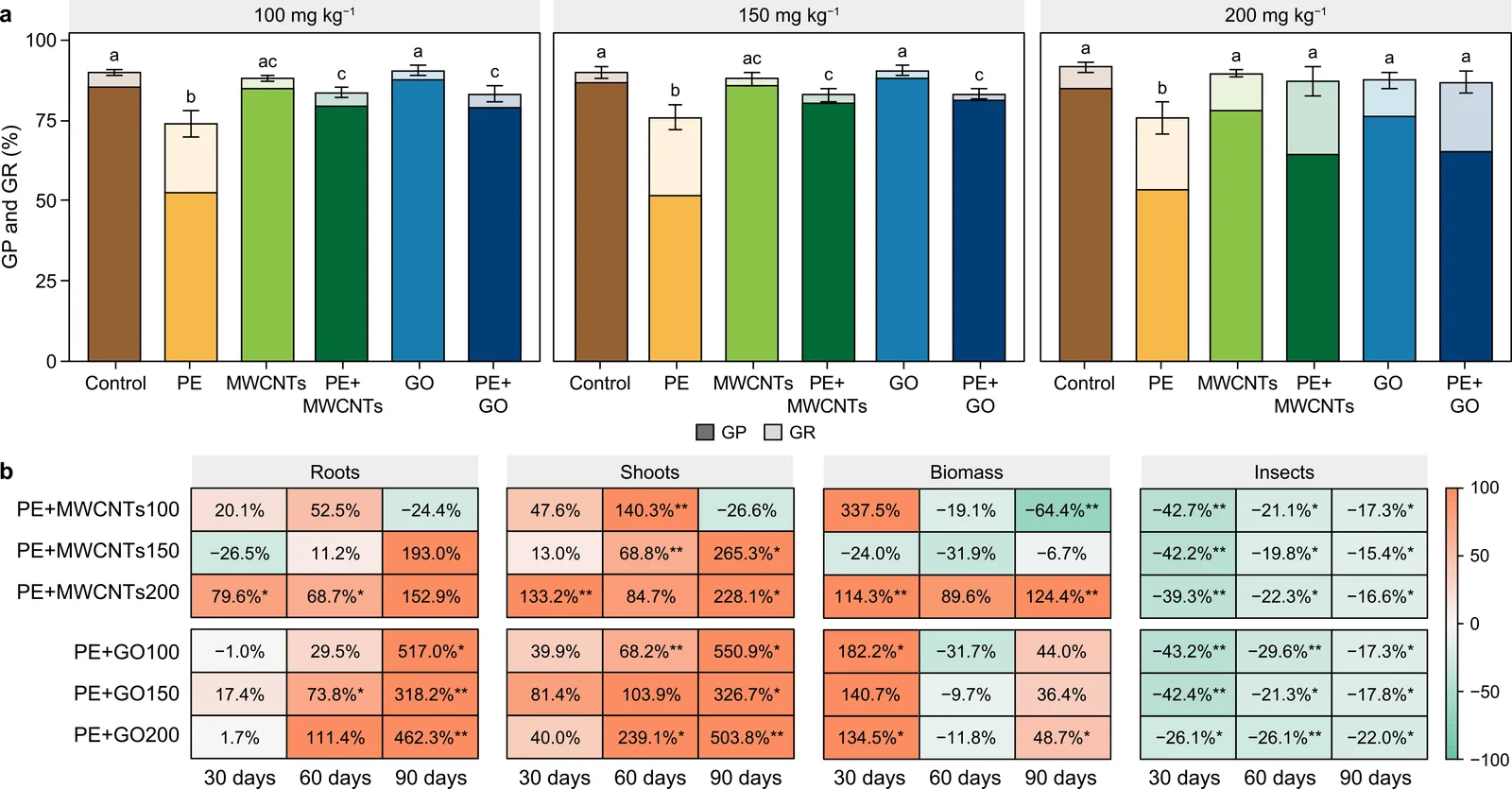
**Germination dynamics and growth responses of alfalfa under different nanomaterial treatments. a**, Germination potential (GP; dark shading) and germination rate (GR; light shading) of seeds exposed to multi-walled carbon nanotubes (MWCNTs) or graphene oxide (GO) at 100, 150, or 200 mg kg^−1^, with or without polyethylene (PE). Bars show means, and error bars show the standard deviation from three independent replicates. Different lowercase letters indicate [please specify the statistical comparison and test]. **b**, Percentage changes in root length, shoot height, biomass, and insect-trap counts after 30, 60, and 90 days relative to the PE + plant. Asterisks indicate significant differences from the PE + plant (∗*p* < 0.05; ∗*∗p* < 0.01). Data for the 150 mg kg^−1^ treatments were obtained from a separate experimental batch; see Materials and Methods for details. 100/150/200 mean 100/150/200 mg kg^−1^.

The effects of MWCNTs and GO on alfalfa growth were evaluated by measuring root length, shoot height, biomass, and insect trap counts at 30, 60, and 90 days. In the PE + plant, root length, shoot height, biomass, and insect counts remained stable across the three time points (Supplementary Fig. S3). These parameters were presented as percentage change relative to the PE + plant (Fig. 2b), with absolute values and statistical groupings provided in Supplementary Fig. S3. Soil pH remained within 7.0 ± 0.5 from 0 to 90 days in all groups. All trapped insects were morphologically identified as fungus gnats (*Bradysia* spp., Sciaridae), a common greenhouse pest whose larvae feed on organic matter and plant roots in moist soil (Supplementary Fig. S2c).

For MWCNT treatments, a clear concentration- and time-dependent pattern emerged. At 100 mg kg^−1^, root length was 20.1% and 52.5% higher than PE + plant after 30 and 60 days, respectively, but 24.4% lower after 90 days, although the latter difference was not significant. Shoot height showed a similar pattern, increasing by 140.4% at 60 days (*p* < 0.01) before falling to 26.6% below PE + plant at 90 days. Biomass was 337.5% higher at 30 days (*p* < 0.01) but 64.5% lower at 90 days. Insect counts were 17.3–42.7% lower across sampling times (*p* < 0.05).

At 150 mg kg^−1^, the relative increase in root length reached 193.0% at 90 days (*p* < 0.01). Shoot height was 265.3% higher than PE + plant, whereas biomass was 6.7% lower, with no significant biomass difference. Insect counts were significantly reduced by 15.4–42.2%. At 200 mg kg^−1^, root length, shoot height, and biomass at 90 days exceeded the PE + plant by 152.9% (*p* < 0.05), 228.1% (*p* < 0.01), and 124.4% (*p* < 0.05), respectively. Insect counts were 16.6–39.3% lower across sampling times (*p* < 0.05).

GO treatments also exhibited pronounced concentration- and time-dependent effects. At 100 mg kg^−1^, root length differed little from the PE + plant at 30 days (−1.0%) and increased by 29.5% at 60 days; neither difference was significant. By 90 days, root length and shoot height were 517.0% and 550.8% higher, respectively (*p* < 0.01), whereas the 44.0% increase in biomass was not significant. Insect counts were consistently reduced by 17.3–43.2% (*p* < 0.05).

At 150 mg kg^−1^, root length and shoot height at 90 days increased by 318.2% and 326.7%, respectively (*p* < 0.01), while biomass increased by 36.4% without a significant difference from PE + plant. Insect counts were reduced by 17.8–42.4% (*p* < 0.05). At 200 mg kg^−1^, root length, shoot height, and biomass at 90 days increased by 462.3% (*p* < 0.01), 503.8% (*p* < 0.01), and 48.7% (*p* < 0.05), respectively. Insect counts were 22.0–26.0% lower, although the difference at 30 days was not significant.

Overall, both MWCNTs and GO promoted alfalfa growth at specific concentrations and time points, with the most pronounced effects observed at 150 and 200 mg kg^−1^ for MWCNTs and at 100 and 150 mg kg^−1^ for GO at 90 days. Insect trap counts were consistently lower in almost all NM-treated groups compared with the PE + Plant.

### 3.3 Nanomaterial effects on PE degradation

#### 3.3.1 Dynamic degradation data of PE

The degradation rates of PE films buried at two depths (surface and bottom) were monitored over 90 days under different NM treatments (Fig. 3a–d). In the planted control without NMs, the degradation rate remained minimal throughout the experiment, reaching only 1.2% in the bottom layer and 0.6% in the surface layer at 90 days. Additionally, in all PE + NM treatments, no detectable degradation was observed at any time point (degradation rate < 0.1%, data not shown in Fig. 3).

**Fig. 3:**
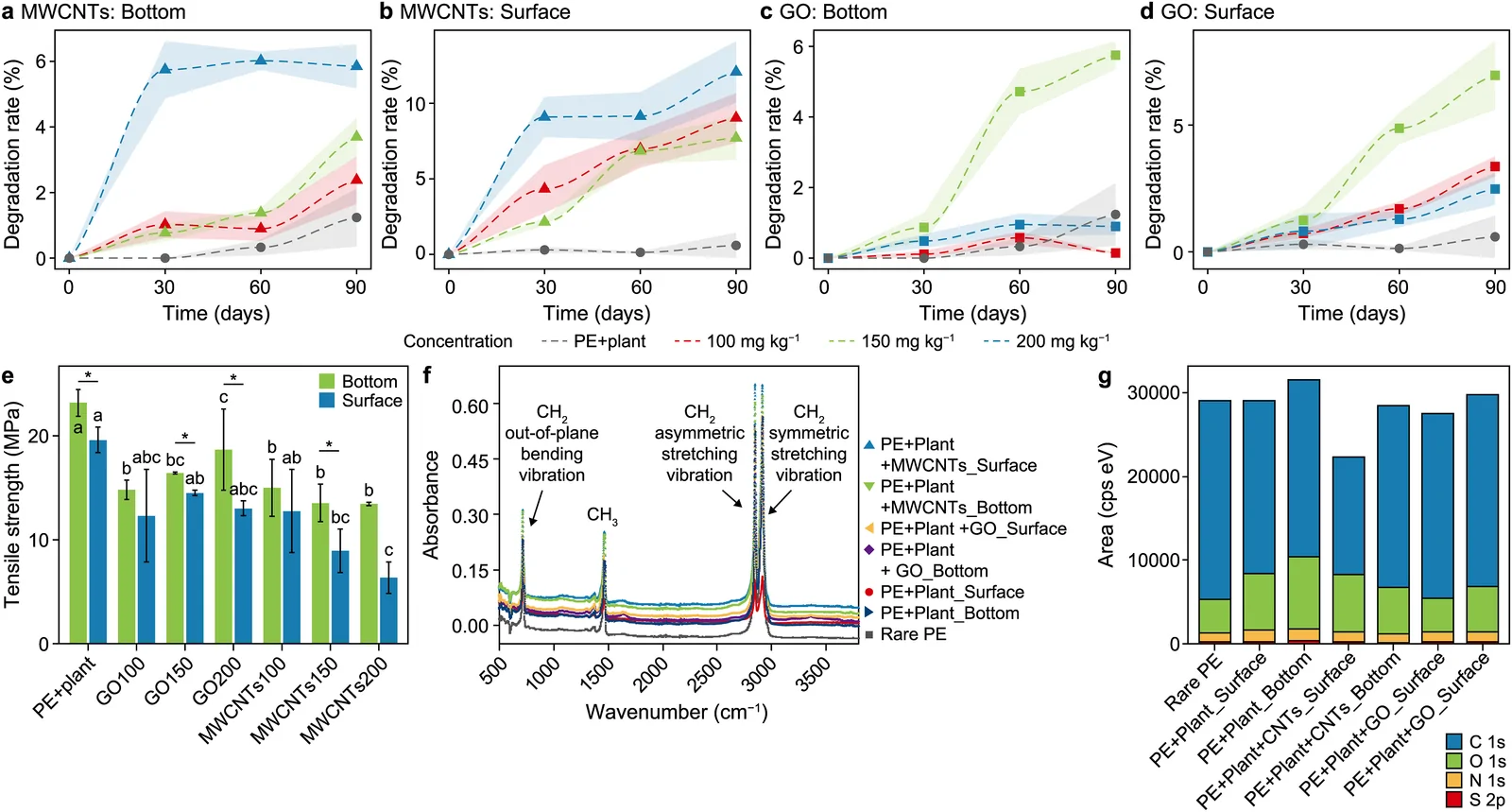
**Degradation of polyethylene films and associated physicochemical changes. a–d**, Changes in the degradation rate of PE films over 90 days in the bottom and surface soil layers amended with MWCNTs (**a**,**b**) or GO (**c**,**d**) at the indicated concentrations. Lines show means, and shaded bands indicate the standard deviation (SD) from three independent replicates. **e**, Tensile strength of PE films recovered from the bottom and surface layers after 90 days. Bars show means, and error bars indicate SD from three independent replicates. Lowercase letters indicate significant differences among treatments within the same soil layer according to Tukey's HSD test (*p* < 0.05). Asterisks above brackets indicate differences between the bottom and surface layers according to two-sided *t*-tests (∗*p* < 0.05; ∗*∗p* < 0.01; ∗∗∗*p* < 0.001). **f**, Fourier-transform infrared spectra of pristine PE and PE films recovered from the indicated treatments and soil layers after 90 days. **g**, Integrated X-ray photoelectron spectroscopy peak areas for C 1s, O 1s, N 1s, and S 2p in the corresponding PE films. Data for the 150 mg kg^−1^ treatments were obtained from a separate experimental batch; see Materials and Methods for details. MWCNTs, multi-walled carbon nanotubes; GO, graphene oxide; PE, polyethylene. Treatments are labeled as PE + Plant + [NM], where “Plant” denotes the presence of alfalfa and “PE” indicates the polyethylene film. Groups without plants are labeled as PE only (no NM) or NM + PE (NM without plants). “Surface” and “Bottom” indicate the soil layer from which the PE film was recovered. 100/150/200 mean 100/150/200 mg kg^−1^.

MWCNTs substantially enhanced PE degradation, and the effects varied with concentration and time. At the bottom layer, the 200 mg kg^−1^ MWCNT treatment exhibited the highest early degradation, reaching 5.8% at 30 days and maintaining around 6.0% at 60 and 90 days. In contrast, the 100 and 150 mg kg^−1^ treatments showed slower but progressive increases, culminating at 2.4% and 3.7% at 90 days, respectively. In the surface layer, all MWCNT concentrations promoted degradation more effectively than in the bottom layer. The 200 mg kg^−1^ treatment again performed best, with degradation rates increasing from 9.1% at 30 days to 12.1% at 90 days. The 100 and 150 mg kg^−1^ treatments also showed steady increases, reaching 9.0% and 7.7% at 90 days, respectively.

GO treatments also accelerated PE degradation, but the effects were generally weaker than those of MWCNTs and more concentration-dependent (Fig. 3c and d). At the bottom layer, only the 150 mg kg^−1^ GO treatment induced substantial degradation, increasing from 0.9% at 30 days to 5.8% at 90 days. The 100 and 200 mg kg^−1^ treatments remained below 1.0% throughout the experiment. At the surface layer, the 150 mg kg^−1^ GO treatment again gave the highest degradation, compared with 3.4% and 2.5% at 100 and 200 mg kg^−1^, respectively.

Overall, both NMs promoted PE degradation, with MWCNTs showing superior efficacy, particularly at the surface layer and at higher concentrations. The 200 mg kg^−1^ MWCNT treatment achieved the highest degradation rate (12.1% at 90 days on the surface), whereas GO was most effective at 150 mg kg^−1^.

#### 3.3.2 Changes in PE strength

The mechanical integrity of PE films was assessed by measuring the elastic modulus after 90 days of exposure (Fig. 3e). For reference, the initial elastic modulus of pristine PE films was 782.8 ± 0.3 MPa, which decreased to 523.8 ± 25.1 MPa after 90 days of burial in soil without plants. The mean elastic modulus for the PE + NM treatments further decreased to 499.0 ± 63.9 MPa, indicating that soil burial and NMs alone can induce plastic aging. However, when combined with alfalfa, the elastic modulus decreased much more substantially across all treatments. In the PE + plant, the modulus was 23.2 MPa in the bottom layer and 19.6 MPa in the surface layer, with a significant difference between depths (*p* < 0.001), indicating greater natural degradation at the surface due to light exposure and rhizosphere activity.

For MWCNT treatments, elastic modulus decreased with increasing concentration at both soil depths, with more pronounced reductions in the surface layer. The lowest values at both depths occurred at 200 mg kg^−1^, and surface-layer films at this concentration had the lowest modulus among all treatments. Compared with the PE + plant, all three MWCNT concentrations significantly reduced the modulus of bottom-layer films (*p* < 0.05), whereas significant reductions in surface-layer films occurred only at 150 and 200 mg kg^−1^. Differences between depths were significant at 150 and 200 mg kg^−1^ but not at 100 mg kg^−1^.

GO produced a different pattern. In the bottom layer, elastic modulus increased with concentration, with the value at 200 mg kg^−1^ significantly exceeding those at 100 and 150 mg kg^−1^ (*p* < 0.05). In the surface layer, the highest modulus occurred at 150 mg kg^−1^. All three GO concentrations significantly reduced the modulus of the bottom-layer films relative to the PE + plant, whereas none of the surface-layer treatments differed significantly from their corresponding controls. Differences between depths were significant at 150 and 200 mg kg^−1^ (*p* < 0.001) but not at 100 mg kg^−1^.

Overall, the elastic modulus measurements corroborated the previously observed degradation patterns (Fig. 3a–d). Treatments with higher degradation rates generally resulted in lower mechanical strength, particularly at the surface layer.

#### 3.3.3 Changes in PE surface and elements

FT-IR spectra were used to examine chemical changes in recovered PE films (Fig. 3f). All samples exhibited characteristic PE absorption bands, including CH_2_ out-of-plane bending at approximately 720 cm^−1^, CH_3_ bending at approximately 1460 cm^−1^, and asymmetric and symmetric CH_2_ stretching at approximately 2920 and 2850 cm^−1^, respectively. Among surface-layer samples, films from the combined plant–MWCNT treatment showed the highest absorbance at these bands, followed by those from the plant–GO treatment and the planted control. In the planted control, bottom-layer films showed higher absorbance than surface-layer films, whereas differences between depths were less pronounced under MWCNT and GO amendment. The broader comparison in Supplementary Fig. S4a also showed greater absorbance in combined plant–NM treatments than in treatments without plants.

Consistent with the FT-IR observations, XPS elemental analysis (Fig. 3g) showed that all samples were dominated by the C 1s signal, corresponding to the carbon backbone of PE. Surface-layer films from the combined plant–MWCNT and plant–GO treatments showed larger O 1s peak areas than pristine PE and the PE-only control. The bottom-layer film from the planted control showed the highest reported O 1s signal. Minor N1s signals were detected in all plant-containing treatments, whereas S 2p signals remained negligible across all groups. This pattern is further supported by the full dataset in Supplementary Fig. S4b, where plant-NM combinations displayed the highest oxygen enrichment compared with both PE + NM and PE-only.

The O 1 s/C 1s peak-area ratio, an indicator of surface oxidation [6], followed a similar trend. The highest ratio was observed in the surface-layer films from the combined plant–MWCNT treatment (0.49), followed by the bottom-layer films from the planted control (0.41) and surface-layer films from the plant–GO treatment (0.18). The corresponding ratio for pristine PE was 0.17.

### 3.4 Spatiotemporal changes of microbial communities

#### 3.4.1 Community diversity and structure

Microbial richness varied with treatment, soil depth, and sampling time (Fig. 4a). The Chao1 richness declined over time in the PE-only treatment. In contrast, the presence of alfalfa maintained higher richness at both depths. The PE + NM treatments also showed declines from 30 to 90 days (Fig. 4a). In the PE-only treatment, Chao1 values decreased from 1634.8 at 30 days to 1380.6 at 90 days. The presence of alfalfa (PE + plant) resulted in higher Chao1 values than PE-only at both time points, with surface and bottom layers reaching 1931.8 and 1920.8 at 30 days, and 1549.3 and 1550.2 at 90 days, respectively. Treatments containing NMs without plants (PE + MWCNTs and PE + GO) showed similar declines, from 2274.4 to 1656.0 and from 2019.4 to 1575.7, respectively. When combined with plants, bottom layers exhibited higher Chao1 values than surface layers at both time points. These differences were greatest at 90 days: bottom- and surface-layer values were 2262.5 and 1950.6 under MWCNT treatment, and 2202.9 and 1560.4 under GO treatment. Shannon and Simpson indicators showed broadly similar patterns (Supplementary Fig. S5).

**Fig. 4:**
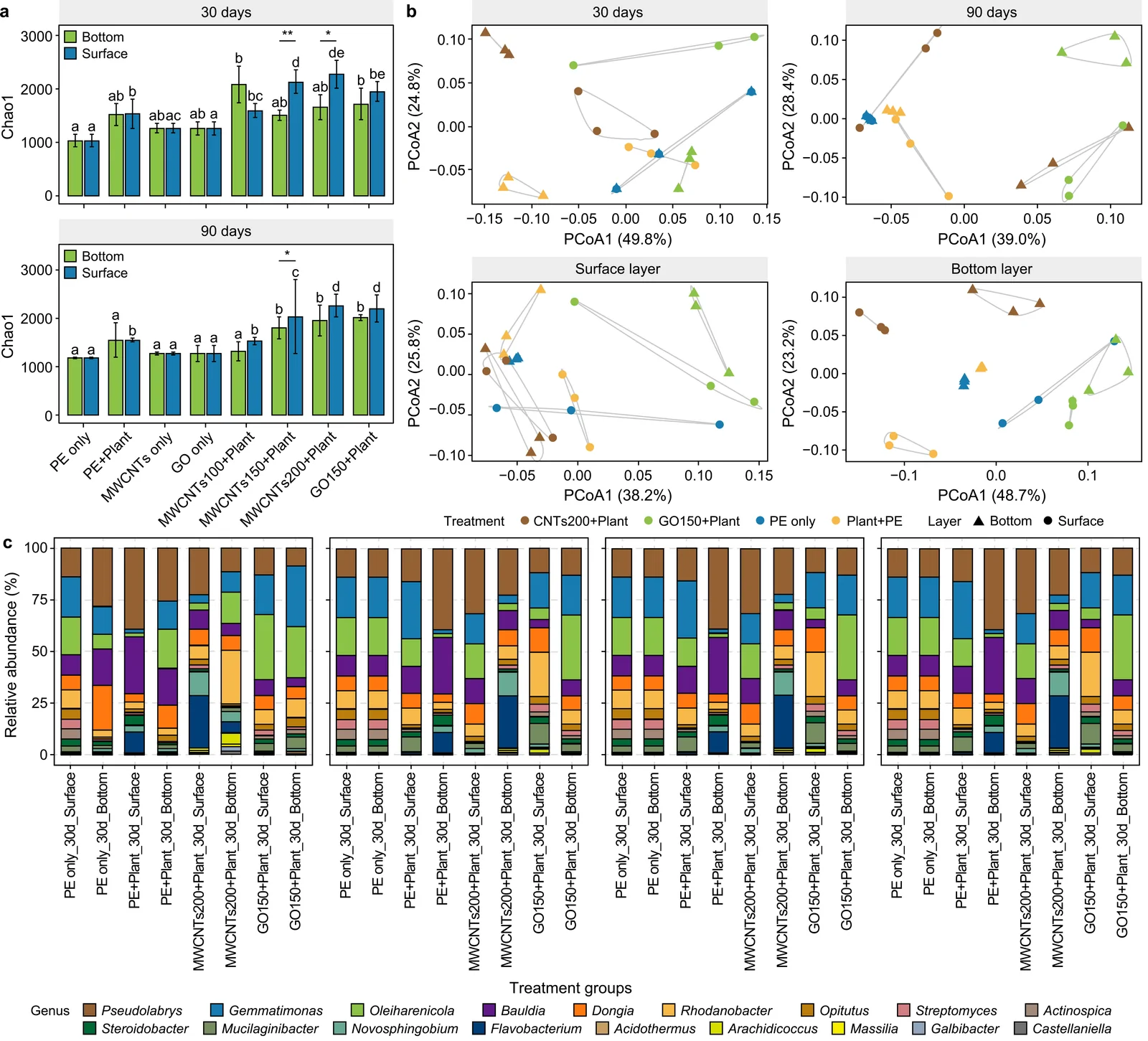
**Diversity, structure, and taxonomic composition of soil microbial communities. a**, Chao1 richness indices after 30 and 90 days in the surface and bottom soil layers under the indicated treatments. Bars show means, and error bars indicate the standard deviation from three independent replicates. Lowercase letters indicate significant differences among treatments within the same depth or time point (Tukey's HSD test, *p* < 0.05). Asterisks above brackets indicate differences between the bottom and surface layers according to two-sided *t*-tests (∗*p* < 0.05; ∗*∗p* < 0.01; ∗∗∗*p* < 0.001). **b**, Principal coordinate analyses (PCoA) based on Bray–Curtis dissimilarities, stratified by sampling time and soil layer. Colors identify treatments, and symbols distinguish surface and bottom samples. Lines connect replicate samples within the same treatment group to facilitate visual differentiation of clusters. Percentages on the axes indicate the variation explained by each coordinate. **c**, Relative abundances of the selected microbial genera across treatments, sampling times, and soil layers. The 30 most abundant genera were selected for display based on their mean relative abundance in the PE-only_30d_Surface samples. Unclassified taxa were excluded. Data for the 150 mg kg^−1^ treatments were obtained from a separate experimental batch; see Materials and Methods for details. MWCNTs, multi-walled carbon nanotubes; GO, graphene oxide; PE, polyethylene. 100/150/200 mean 100/150/200 mg kg^−1^.

Principal coordinate analysis (PCoA) revealed differences in microbial community structure among treatments, depths, and sampling times (Fig. 4b). The 30-day PCoA highlighted the dominant role of rhizosphere status, with surface (rhizosphere) and bottom (non-rhizosphere) samples forming distinct clusters. At this time point, the plant-NM treatments had already diverged from the PE-only group in the surface layer, whereas the PE + plant and PE-only groups remained closely clustered. By 90 days, samples from the PE + plant group converged across layers, indicating community homogenization, while the plant-NM treatments still maintained clear separation. Over time, all planted treatments, including the PE + plant and treatments amended with 200 mg kg^−1^ MWCNTs or 150 mg kg^−1^ GO, became more distinct from the PE-only control along PCoA1. Temporal changes also differed between depths. In the surface layer, samples from 30 to 90 days generally clustered together within each treatment, with almost complete overlap in the planted treatment amended with 200 mg kg^−1^ MWCNTs. In the bottom layer, samples from the two time points were clearly separated within each treatment.

#### 3.4.2 Abundance dynamics of key microbial genera and co-occurrence networks

The relative abundances of microbial genera varied among treatments, soil depths, and sampling times (Fig. 4c). For bottom samples between 30 and 90 days, clear differences were observed between the experimental groups and the PE-only group. Under the planted treatment amended with 200 mg kg^−1^ MWCNTs, the bottom community underwent substantial changes, characterized by a marked increase in the abundance of multiple genera. Changes in the planted treatment amended with 150 mg kg^−1^ GO were smaller, with the overall abundance ranking largely preserved. In the surface layer, the experimental groups also differed from the PE-only group over the 30–90-day period, but temporal changes within each treatment were comparatively limited. Focusing on day 30, pronounced differences between surface and bottom communities were evident across all experimental groups, with the most abundant genera differing between the two layers. This phenomenon persisted at day 90. Using the two NM-amended groups as examples, *Rhodanobacter* dominated the bottom-layer community under 200 mg kg^−1^ MWCNT amendment and the surface-layer community under 150 mg kg^−1^ GO amendment, whereas *Oleiharenicola* dominated the opposite layer in each treatment.

Clustering of the 15 most abundant genera further illustrated these spatial and temporal differences (Fig. 5a). At day 30, the bottom and surface samples of the 200 mg kg^−1^ MWCNT treatment showed clear clustering separation and exhibited substantial differences in microbial composition, although most of these differences were not statistically significant, except for *Novosphingobium*. A similar pattern was observed for the 150 mg kg^−1^ GO amendment, where *Arachidicoccus* was significantly more abundant on the surface than at the bottom. The Plant-only group exhibited the greatest clustering divergence between the surface and bottom, indicating widespread community differences between the two layers. By day 90, in the 200 mg kg^−1^ MWCNT treatment, only *Bauldia* was significantly enriched at the surface compared with the bottom, whereas in the 150 mg kg^−1^ GO amendment, both *Oleiharenicola* and *Opitutus* showed significantly higher abundances at the surface. All experimental groups clustered away from the PE-only control, underscoring the impact of plant and NM amendments.

**Fig. 5:**
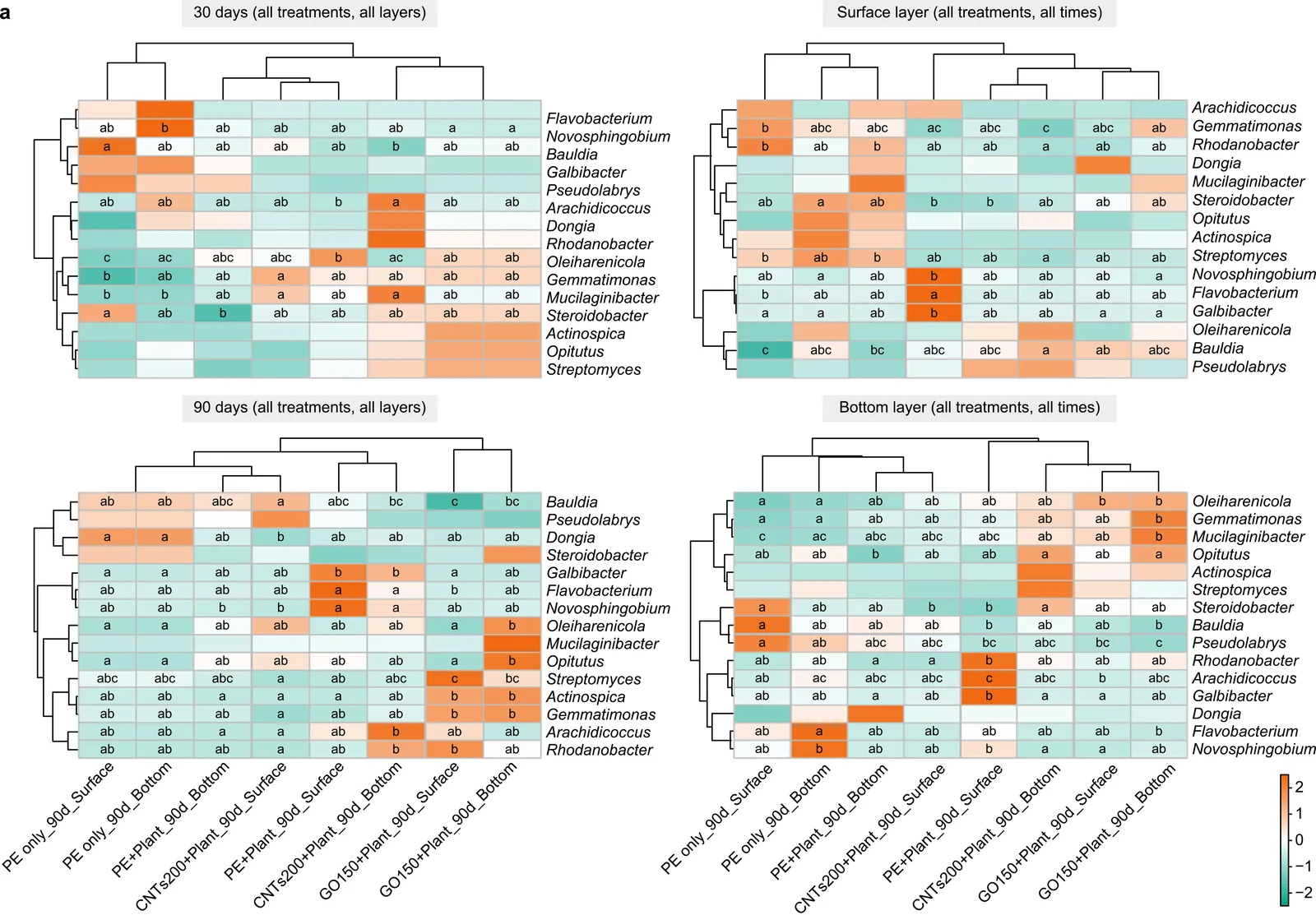

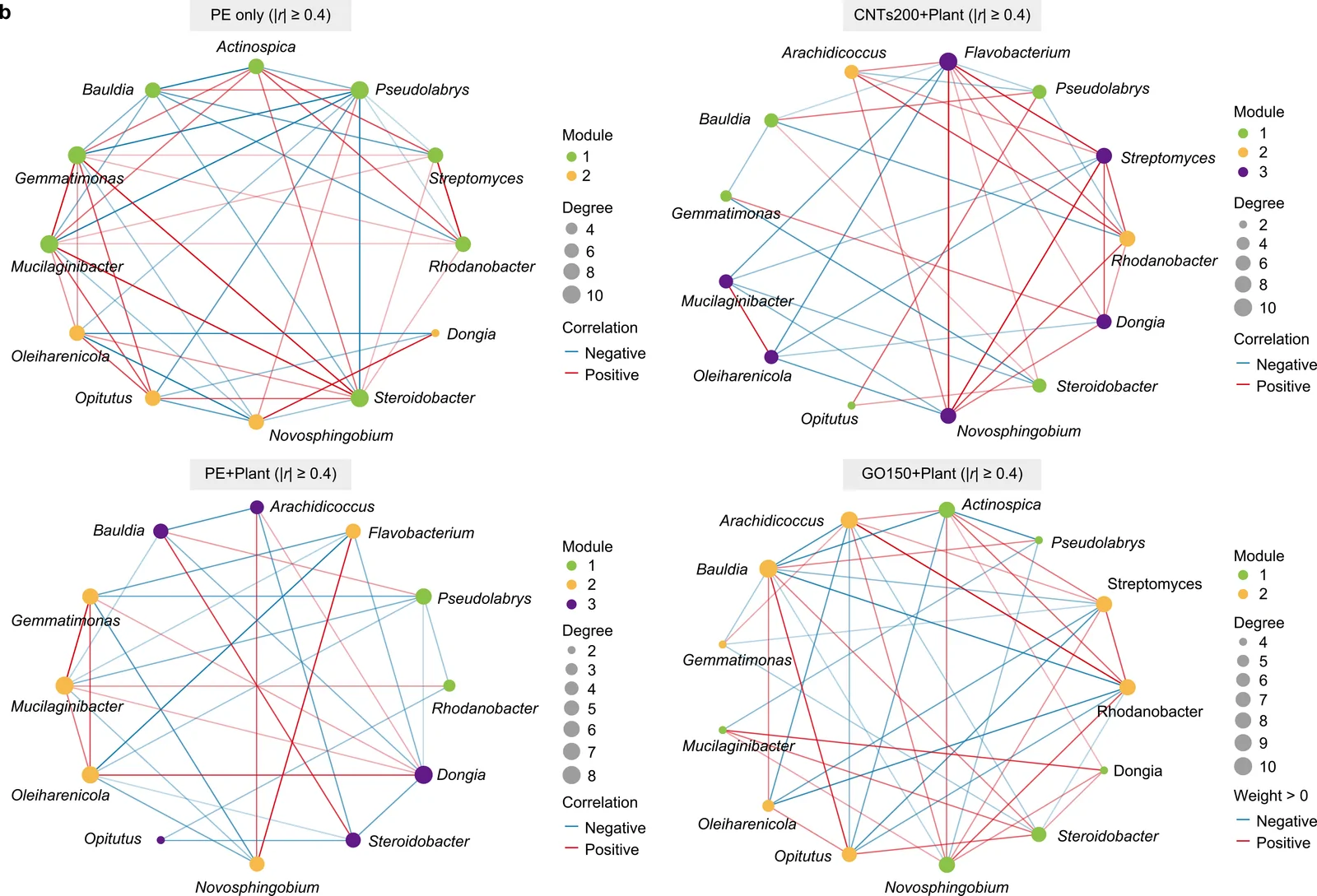
**Treatment-related variation and co-occurrence patterns of dominant microbial genera. a**, Hierarchically clustered heatmaps showing normalized abundances of dominant genera across treatments, sampling times, and soil layers. Colors represent standardized relative abundances according to the adjacent scale. Lowercase letters indicate significant differences among treatments within the same depth or time point (Tukey's HSD test, *p* < 0.05). **b**, Co-occurrence networks for the PE-only, plant + PE, MWCNTs200 + plant, and GO150 + plant treatments. Node colors identify network modules detected by modularity analysis; node sizes represent degree (number of connections per node). Edge colors indicate positive (red) or negative (blue) correlations, and edge transparency is proportional to the absolute Spearman correlation strength (|*r*|). Only associations with |*r*| ≥ 0.4 and adjusted *p* < 0.05 (Benjamini-Hochberg) are shown. Data for the 150 mg kg^−1^ treatments were obtained from a separate experimental batch; see Materials and Methods for details. MWCNTs, multi-walled carbon nanotubes; GO, graphene oxide; PE, polyethylene. 100/150/200 mean 100/150/200 mg kg^−1^.

In the surface layer, the dominant genera under the 200 mg kg^−1^ MWCNT treatment remained consistent from day 30 to day 90 (*Bauldia*, *Oleiharenicola,* and *Pseudolabrys*). The corresponding 150 mg kg^−1^ GO treatment showed higher relative abundances of *Gemmatimonas* and *Rhodanobacter*. In the bottom layer, dominant genera under 200 mg kg^−1^ MWCNT amendment underwent a marked shift between day 30 and day 90, with the dominant taxa changing from *Flavobacterium* and *Novosphingobium* to *Rhodanobacter*, *Arachidicoccus,* and *Galbibacter*, and the clustering analysis confirmed this temporal differentiation. In contrast, the bottom-layer community under 150 mg kg^−1^ GO amendment remained relatively stable over time and clustered closely with the day 30 PE-only sample, indicating that its composition was largely reinforced rather than restructured.

Co-occurrence networks based on Spearman correlations (|*r*|≥0.4) revealed how microbial interactions were reshaped by different treatments (Fig. 5b). In the PE-only network, large nodes (high degree) were occupied by *Actinospica*, *Pseudolabrys*, *Gemmatimonas*, and *Rhodanobacter*, with two modules and a slight dominance of positive correlations. The PE + plant network introduced a third module and increased the proportion of positive edges. Key hubs included *Flavobacterium*, *Dongia*, and *Mucilaginibacter*. The planted treatment amended with 200 mg kg^−1^ MWCNTs maintained three modules and a balanced positive/negative ratio, with *Flavobacterium*, *Streptomyces*, and *Mucilaginibacter* as hubs, showing a more compartmentalized yet stable interaction web. The planted treatment amended with 150 mg kg^−1^ GO exhibited the highest proportion of positive correlations and only two modules, with *Rhodanobacter*, *Streptomyces*, and *Actinospica* as central nodes, showing strong functional convergence and synergistic interactions. Positive-edge density increased from the PE-only control to the planted control without NMs, followed by the planted treatments amended with 200 mg kg^−1^ MWCNTs and 150 mg kg^−1^ GO. These network changes accompanied enhanced PE degradation, suggesting a potential link between microbial community associations and degradation performance. The Venn diagrams support the evidence of community changes (Supplementary Fig. S6).

#### 3.4.3 Functional profiling of the microbial community

Predicted abundances of Kyoto Encyclopedia of Genes and Genomes (KEGG) orthologs (KOs), standardized as *Z*-scores, were compared among treatments, soil depths, and sampling times (Fig. 6). Functional descriptions and database links for the selected KOs are provided in Supplementary Table S3.

**Fig. 6:**
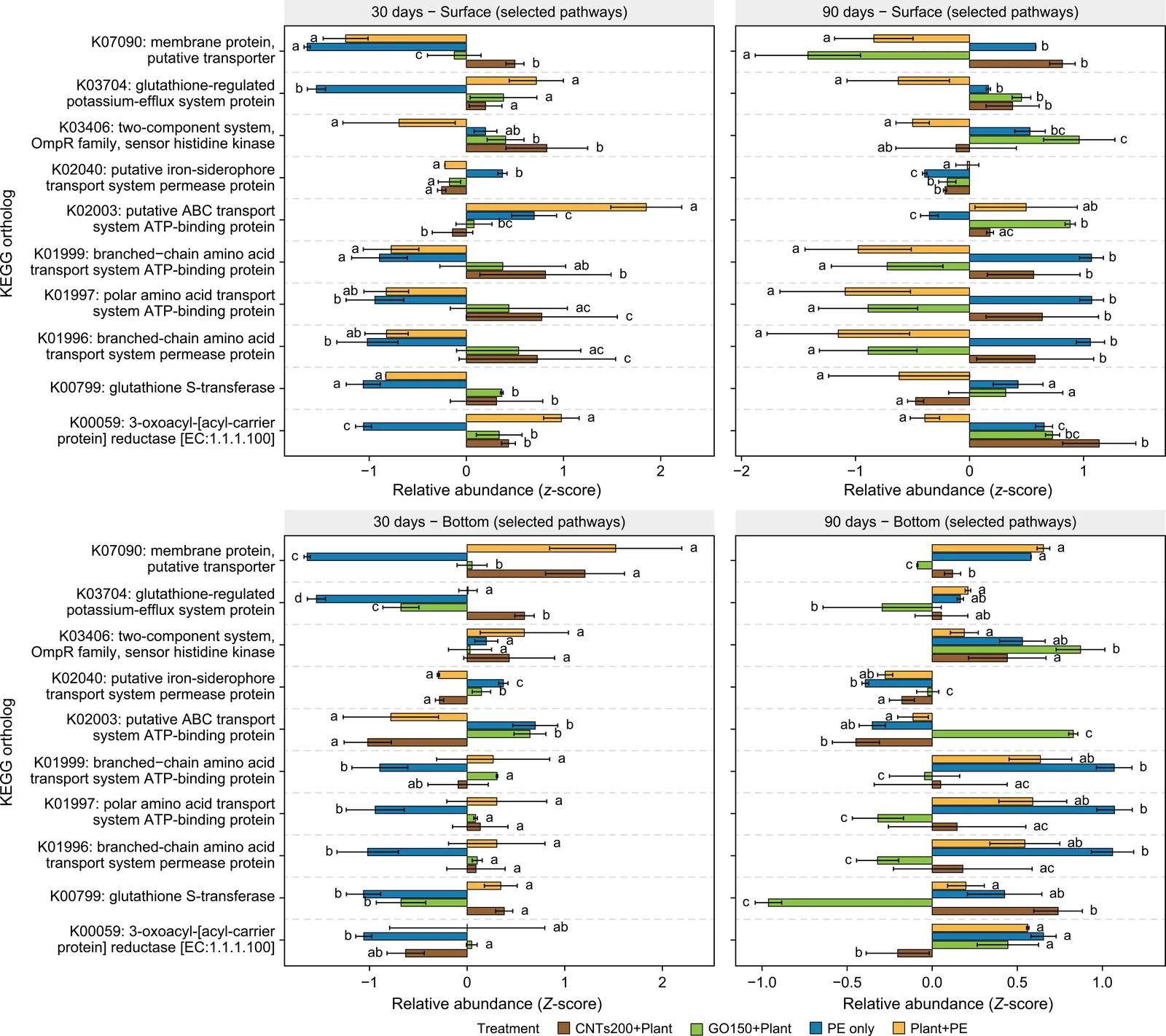
**Relative abundances of selected KEGG orthologues under different treatments**. *Z-*score-normalized relative abundances of selected KEGG orthologues in the surface and bottom soil layers after 30 and 90 days. Bars show means, and error bars indicate the standard deviation from three independent replicates. Lowercase letters indicate significant differences among treatments within the same depth or time point (Tukey's HSD test, *p* < 0.05). Colors identify the treatment groups. The criteria used to select the displayed KEGG orthologues should be specified. Data for the 150 mg kg^−1^ treatments were obtained from a separate experimental batch; see Materials and Methods for details. KEGG, Kyoto Encyclopedia of Genes and Genomes; PE, polyethylene; TCA, tricarboxylic acid. 100/150/200 mean 100/150/200 mg kg^−1^.

In the surface layer on day 30, plant-associated pathways, such as amino acid and ABC transporters, were most enriched in the PE + plant treatment. By day 90, the PE-only and the planted treatment amended with 200 mg kg^−1^ MWCNTs showed the highest abundances of lipid metabolism-related genes (e.g., 3-oxoacyl-ACP reductase K00059) and general transporter systems. In the bottom layer, the day 30 samples were dominated by PE + plant with moderate expression of stress-related genes (e.g., glutathione S-transferase K00799), whereas at day 90, the PE-only and the planted treatment amended with 200 mg kg^−1^ MWCNTs exhibited markedly elevated *Z*-scores (>1) for amino acid and peptide transport pathways.

These temporal changes were accompanied by differences between depths. At day 30, the surface layer exhibited higher functional activity (especially in PE + plant) than the bottom layer, with all treatments remaining near the baseline. By day 90, the two layers converged, both showing the highest activities in PE-only and the planted treatment amended with 200 mg kg^−1^ MWCNTs. Statistical letters [Tukey's honestly significant difference (HSD) test, p < 0.05] confirmed that at day 30, significant differences were confined to the surface (e.g., PE + plant with letter a for K00799), while at day 90, the bottom layer acquired distinct high-letter groups (e.g., PE-only and the planted treatment amended with 200 mg kg^−1^ MWCNTs with a or b for several amino acid transporters).

Among the ten selected KOs, the largest fold increases in the planted treatment amended with 200 mg kg^−1^ MWCNTs relative to controls were observed for K00059, K00799, K02003, K02040, K03704, K07090, K03406, K01996, K01997, and K01999, pointing to enhanced fatty acid metabolism, detoxification, iron uptake, and amino acid transport in the NM-amended rhizosphere. Based on KEGG data, a potential degradation pathway was hypothesized, involving sequential C–C bond cleavage, detoxification, and potential entry into the tricarboxylic acid cycle. This pathway links the key functional genes identified in the NM-amended rhizosphere to the ultimate mineralization of PE-derived intermediates.

#### 3.4.4 Correlation network linking plant traits, microbial taxa, functional genes, and degradation

Correlation analyses examined relationships among plant growth traits, soil properties, microbial genera, functional genes, and the PE degradation rate in MWCNT-amended treatments (Fig. 7). These analyses included only the microorganisms (Fig. 5) with plastic-degrading or antagonistic potential and the KEGG pathways significantly enriched in the MWCNT group (Fig. 6).

**Fig. 7:**
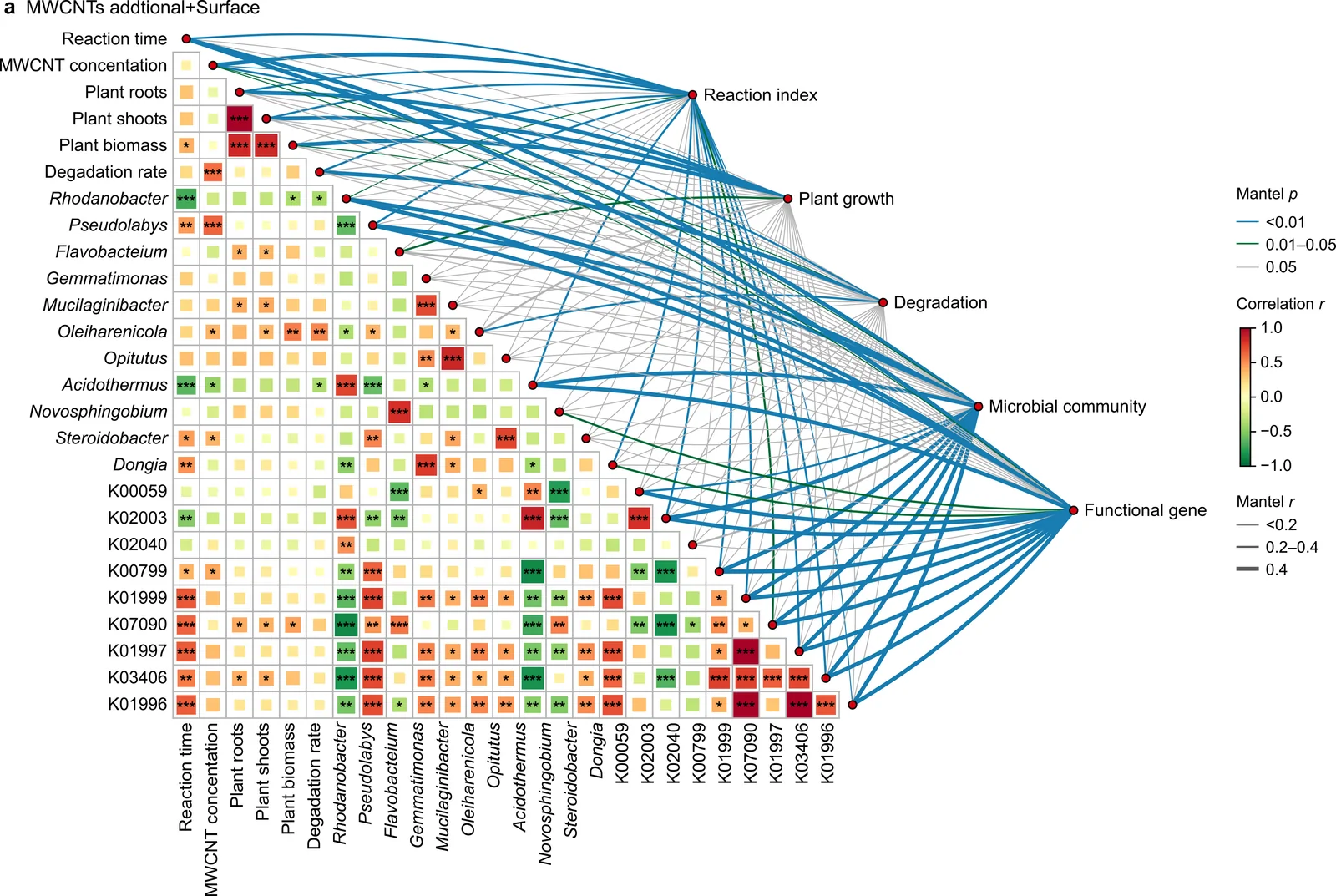

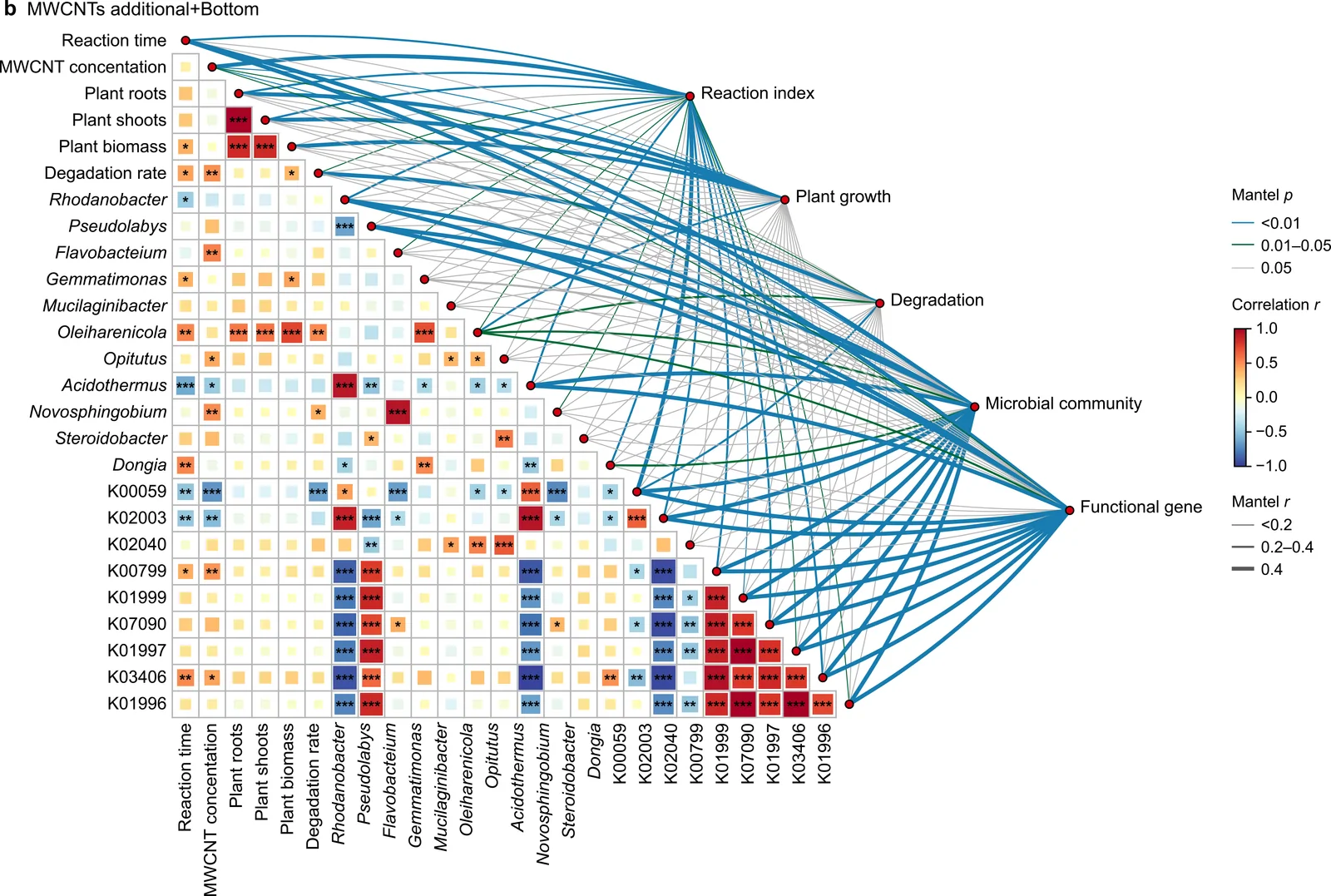
**Associations among experimental conditions, plant traits, microbial communities, functional genes and polyethylene degradation under MWCNT amendment**. Mantel-test correlation networks for the surface (**a**) and bottom (**b**) soil layers, incorporating the MWCNTs100 + plant, MWCNTs150 + plant, and MWCNTs200 + plant treatments. The correlation matrices show pairwise correlation coefficients, with square color and size indicating the direction and magnitude of the association and asterisks indicating statistical significance. Network-edge colors indicate Mantel-test *p-*values, and edge widths indicate Mantel *r* values, as defined in the legends. Grey edges represent non-significant associations. MWCNTs, multi-walled carbon nanotubes. 100/150/200 mean 100/150/200 mg kg^−1^.

The list of microorganisms is presented in Supplementary Table S2. In the surface layer, the network exhibited dense positive correlations (Fig. 7a). NM concentration and reaction time were positively correlated with *Pseudolabrys*. Plant growth indices were strongly and positively linked with *Flavobacterium*, *Mucilaginibacter*, and *Oleiharenicola*, as well as with PE degradation rate. Most KEGG orthologs (e.g., K07090 and K03406) also exhibited positive correlations with reaction time, plant growth, and the majority of potential plastic-degrading genera, except for *Acidothermus* and *Novosphingobium*. However, these two genera were strongly correlated with K02003 and K07090, respectively. Mantel analysis indicated that the reaction parameters (concentration and time) were significantly associated with both the microbial community and functional genes, especially reaction time. Plant growth showed no significant associations with other microbial genera or functional genes, except *Flavobacterium*. Degradation rate was significantly associated only with *Oleiharenicola* and reaction time. At the bottom layer, the overall correspondence between microbial genera and functional genes was similar to that observed in the surface layer (Fig. 7b). K02003 showed a strong positive correlation with *Rhodanobacter*. Among the plant growth indicators, *Oleiharenicola* remained significantly correlated, while *Gemmatimonas* replaced the other genera. *Oleiharenicola* was also the only genus significantly associated with the degradation rate. MWCNT concentration significantly enhanced the correlations with K00799 and K03406.

## 4 Discussion

Studies on plant-assisted plastic degradation remain limited, with reported mass losses of below 3% for PE and approximately 8% for PBAT [11,12]. Quantitative evidence from other plant-based systems is scarce, and few studies have examined the additional effects of externally applied nanomaterials [10,25]. This study demonstrates that commercial MWCNTs substantially enhance PE degradation in an alfalfa-soil system, achieving a surface degradation rate of 12.08% within 90 days, which is significantly higher than previous plant-only results. Beyond mass loss, FT-IR and XPS analyses indicated surface oxidation of the PE films (Fig. 3 and Supplementary Fig. S4). Together with the KEGG functional predictions, these findings support the proposed degradation pathway. These findings set the stage for a deeper discussion of the spatiotemporal mechanisms underlying NM-assisted rhizoremediation. The proposed framework summarises the protective effects of NMs on alfalfa and their promotion of PE degradation (Fig. 8).

**Fig. 8:**
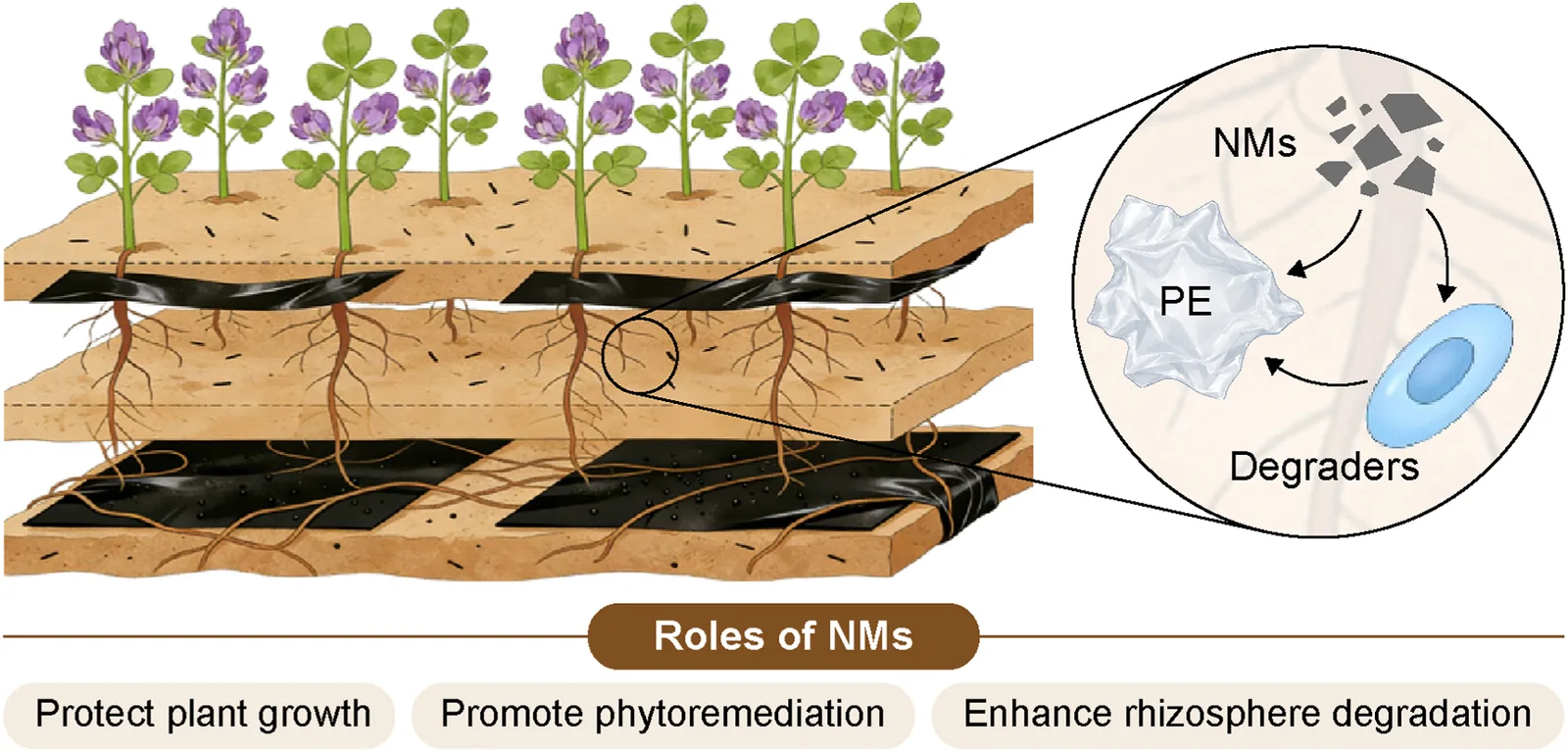
**Proposed mechanisms underlying nanomaterial-assisted polyethylene degradation and plant protection.** Schematic illustrating the proposed effects of NMs on plant growth, phytoremediation, and rhizosphere-associated PE degradation. NMs, nanomaterials; PE, polyethylene.

### 4.1 Contrasting modes of action: MWCNTs as early catalyst-like acceleration vs. GO as sustained rhizosphere regulators

In contrast to the negligible degradation (0.6–1.2% over 90 days) observed in unamended controls, both NMs accelerated the breakdown of PE. MWCNTs showed a rapid initial effect, whereas GO promoted a gradual, sustained increase, especially in the bottom layer. This divergence can be attributed to the physicochemical properties of each material. MWCNTs, with their abundant surface functional groups and high specific surface area, likely facilitate early radical generation and direct polymer cleavage [5,14], as suggested by the rapid enrichment of *Pseudolabrys* and the early activation of K00799 (glutathione S-transferase). MWCNT-amended surface films exhibited the strongest increase in O 1s signal and CH_2_ stretching absorbance (Fig. 3f and g), indicating that the rapid mass loss was accompanied by surface oxidation and chemical changes related to chain breakage [5,22].

However, the tendency of MWCNTs to aggregate in soil matrices and their lower biocompatibility may limit their long-term efficacy [10,26], as reflected in the plateaued degradation after 30 days. In contrast, GO, characterized by a stable lamellar structure and continuous release of oxygen-containing functional groups [27,28], appears to exert a more sustained influence on the rhizosphere microbiome. The progressive enrichment of *Rhodanobacter* and the sustained activation of K02003 (iron-siderophore transporter) in GO treatments align with a mechanism of gradual, microbially mediated degradation rather than direct catalyst-like acceleration. This interpretation is consistent with the observed positive feedback between GO-enhanced plant growth and bottom-layer degradation, suggesting that GO promotes a self-reinforcing rhizosphere process [10,29]. The mechanical integrity of the recovered PE films, assessed by elastic modulus measurements, corroborated the degradation trends. For instance, the planted treatment amended with 200 mg kg^−1^ MWCNTs resulted in the greatest reduction in elastic modulus, particularly in the surface layer, consistent with its highest degradation rate, whereas the planted treatment amended with 150 mg kg^−1^ GOs exhibited a more moderate loss of strength that aligned with its sustained but lower overall degradation, consistent with findings in bio-recyclable materials [30].

### 4.2 Ecological implications of spatiotemporal dynamics: surface vs. bottom

The vertical stratification of degradation activity underscores the distinct ecological niches within the soil–plant continuum, (Fig. 3). Unlike previous studies that focused on bulk soil or single-depth analysis, this work explicitly distinguishes between the surface and bottom layers, revealing that the rhizosphere effect on PE degradation is both depth-dependent and temporally dynamic. In the surface layer, direct photodegradation is unlikely because the PE films were buried 2 cm below the soil surface. Instead, the higher degradation in the surface layer is attributed to greater rhizodeposition and higher activity of aerobic, early-colonizing taxa such as *Pseudolabrys* and *Flavobacterium* [31,32]. Consistently, the PE films from plant-NM co-treatments showed polymer oxidation at the surface layer (Fig. 3). This aligns with evidence that legume rhizospheres accelerate the breakdown of large plastic particles [11].

Conversely, the bottom layer, which becomes rhizosphere only after 60 days, presents a more complex, root-dominated environment where degradation is intimately coupled with microbial metabolism and root exudation [19,20]. Although oxygen fluctuations may occur at this depth, our data revealed a significant increase in aerobic bacteria [33] and no enrichment of anaerobic pathways according to KEGG analysis. The higher and sustained degradation in the GO-amended bottom layer suggests that GO is particularly well suited to harnessing the rhizosphere effect, potentially by mediating electron transfer or providing a stable substrate for microbial colonization [15,34,35]. These findings suggest two distinct application strategies, with MWCNTs offering a clear advantage for rapid, surface-oriented intervention, whereas GO may serve as a secondary option for promoting longer and deeper soil remediation in synergy with plant roots.

### 4.3 Spatiotemporal succession of the microbial community and functional implications

The observed shifts in microbial community structure and function provide a mechanistic basis for differential degradation patterns. In MWCNT treatments, the early enrichment of *Pseudolabrys* and *Oleiharenicola*, coupled with the activation of K00799 (glutathione S-transferase), points to a community geared toward initial oxidative cleavage and detoxification of PE-derived intermediates [36,37]. This aligns with the role of these genera in early carbon utilization and stress response [33]. The subsequent decline in the degradation rate of MWCNTs may reflect the transient nature of this pioneer community. This early recruitment of functional taxa enabled MWCNT-amended communities to establish a stable compositional structure by day 30, which persisted through day 90 (Supplementary Fig. S6). This temporal stability likely provides a consistent functional basis for sustained degradation [13,38], in contrast to the more gradual community reorganization observed in GO treatments.

In GO treatments, the sustained dominance of *Rhodanobacter* and *Gemmatimonas* in the bottom layer, along with the pronounced activation of K02003 and K00059, may indicate a community adapted to the mineralization of degradation products and the acquisition of iron, a critical cofactor for oxygenases involved in hydrocarbon metabolism [38,39]. The strong association of *Gemmatimonas* with nitrogen cycling further suggests that GO-amended systems may foster a more complete, nutrient-coupled degradation process [40,41]. The PE films recovered from the bottom layer of planted GO treatments also displayed elevated O 1 s/C 1s ratios (Fig. 3f), suggesting that the sustained microbial activity promoted by GO led to progressive polymer oxidation even in deeper soil. The potential degradation pathway can be simplified based on the existing evidence as follows: C–C bond cleavage (K00059) → intermediate detoxification (K00799) → transmembrane transport (K02003) → tricarboxylic acid cycle. This is consistent with the temporal and spatial activation of these genes and the co-occurrence of specific microbial taxa [42].

### 4.4 Synergistic mechanisms of plant–microbe–NM interactions

The strong positive correlation between plant growth indices and degradation rate in the NM-amended groups highlights the central role of plants in this tripartite system. No degradation was detected in the PE + NM treatments, underscoring the importance of plants in initiating the degradation cascade. The protective effect of NMs on plant performance was evident from the germination stage onward. While PE alone significantly reduced germination potential and germination rate, similar to the effects of other organic pollutants such as polycyclic aromatic hydrocarbons (PAHs) [32], the addition of MWCNTs or GO alleviated this inhibition. Moreover, plant growth parameters were markedly enhanced by both NMs, particularly in the planted treatment amended with 200 mg kg^−1^ MWCNTs and the planted treatment amended with 150 mg kg^−1^ GOs. These growth improvements not only reflect reduced phytotoxicity [10] but also contribute to a more robust rhizosphere environment [43], thereby reinforcing the positive feedback loop between plant activity and microbial degradation [44].

In contrast to aquatic plants, which primarily adsorb microplastics without true degradation [45], our terrestrial alfalfa system, particularly when combined with NMs, resulted in actual polymer chain cleavage. Alfalfa likely serves a dual function: on the one hand, it is a source of rhizodeposits that fuel microbial activity (e.g., XPS detection of minor N 1s signals in all plant-containing treatments); on the other hand, it acts as a biological pump that translocates water and nutrients, thereby shaping the rhizosphere microbiome [8,10,46]. In the MWCNT treatment, the initial burst of degradation may be partly attributable to NM-induced oxidative stress, which can stimulate root exudation of phenolic compounds and organic acids, further enriching early colonizing degraders [26,47]. The emergence of negative correlations in the MWCNT network may reflect niche differentiation or resource competition among functionally similar taxa, a pattern often observed in stable, structured communities [39]. However, this effect may be transient. In the GO treatment, the stable lamellar structure may facilitate a more favorable redox environment for microbial respiration, as suggested by the predicted enrichment of genes involved in oxidative stress response (K03704, K00799) and iron acquisition (K02003, K02040), thereby potentially promoting the co-metabolic degradation of PE intermediates [34,48]. The resultant release of small organic molecules from PE breakdown could, in turn, be assimilated by plants, creating a carbon loop that sustains both partners [6,49,50]. This mutualistic reinforcement may explain the positive feedback between plant growth and degradation in NM-amended systems. Overall, these results reveal that NMs not only accelerate PE degradation but also actively protect plant performance across the growth cycle, from germination to harvest. This extends beyond the plastic-removal focus of conventional studies [8,51], highlighting a more integrated plant-NM synergy.

### 4.5 Environmental implications and risk–benefit assessment

The contrasting behaviors of MWCNTs and GO offer distinct practical applications. MWCNTs, with their rapid catalyst-like action, are well suited for short-term, intensive remediation of surface-contaminated hotspots, whereas GO, which promoted a more sustained plant-driven process at 150 mg kg^−1^ in the later batch, may merit further investigation for long-term soil restoration [51,52]. However, NM amendment also reduced insect abundance, indicating non-target toxicity, and dose optimization is critical [26,53]. Regarding worker safety, the dilute soil application (≤200 mg kg^−1^) minimizes aerosolization, and standard engineering controls can address concerns related to the asbestos-like morphology of MWCNTs. Cost remains a barrier, but industrial-scale production of carbon NMs is expanding rapidly [54,55], making future cost reductions likely and improving the circular economy [56]. Hence, the approach is potentially viable for targeted applications. Further discussion of risk–benefit, worker safety, and cost is provided in Supplementary Text, Section IV.

Future research should combine metabolomics, root exudate analysis, and enzyme activity assays to definitively map the proposed degradation pathways [57,58]. As demonstrated in algae-based systems, plants and their associated microorganisms can produce enzymes capable of plastic degradation [9], a mechanism that deserves further exploration in terrestrial legume–rhizosphere systems. Furthermore, the long-term ecological fate of these NMs, particularly the potential for MWCNTs to persist and for GO's layered structure to affect soil aggregation, warrants thorough investigation before widespread field application [59,60]. From a life cycle assessment perspective, future evaluations should balance multiple factors, including degradation efficiency and soil health [7], material types and local demand [26], and economic investment [2]. In addition, commercial plastic films contain various additives. For example, the PE film used in this study contained carbon black. The synergistic toxicity of these additives or other pollutants with PE also demands careful attention in future investigations [31,61].

## 5 Conclusion

This study systematically elucidates the temporal (30, 60, and 90 days) and spatial (surface and bottom) regulatory effects of MWCNTs and GO on polyethylene degradation in a soil–plant system. MWCNTs functioned as early-stage catalyst-like agents, achieving a 12% mass loss in the surface layer as a proof of concept for initiating the breakdown of recalcitrant polymers, particularly at 200 mg kg^−1^. Meanwhile, GO played a supporting role in bottom-layer degradation, with the 150 mg kg^−1^ treatment, tested in a later experimental batch, showing promising activity that warrants further validation in future dose–response studies. Both NMs alleviated PE phytotoxicity, as evidenced by improved plant growth indicators relative to the PE-only control, thereby sustaining a functional rhizosphere for prolonged degradation. The spatiotemporal succession of the microbial community and functional genes was identified as a core driver of these contrasting degradation patterns. MWCNTs enriched early degraders, such as *Pseudolabrys* and *Oleiharenicola*, and K00799 showed enrichment in functional predictions, reflecting the rapid recruitment of functional taxa that established a stable community profile from 30 to 90 days. In contrast, GO fostered the sustained presence of degraders, including *Rhodanobacter* and *Gemmatimonas,* and predicted enrichment of K02003, suggesting a more gradual but persistent functional shift that became pronounced only after root colonization of the bottom layer. Together with the upregulation of key functional genes, these findings support a possible PE degradation pathway. By explicitly linking the distinct spatiotemporal behaviors of two commercial NMs to their effects on plant protection and microbial succession in surface and bottom layers, this study provides a framework for integrating carbon NMs into rhizoremediation strategies. These findings highlight the potential of commercial carbon NMs for scalable applications in plastic remediation and provide a basis for optimizing their timing, placement, and concentration. Future studies should focus on the environmental fate of degradation intermediates to experimentally validate the proposed C–C cleavage and detoxification pathway, as well as on the long-term ecological effects of the NMs themselves to ensure the safety and sustainability of large-scale applications.

## CRediT authorship contribution statement

**Haoran Liu:** Writing – original draft, Visualization, Validation, Software, Resources, Project administration, Methodology, Investigation, Formal analysis, Data curation, Conceptualization. **Lena Ciric:** Writing – review & editing, Supervision. **Manpreet Bhatti:** Writing – review & editing, Writing – original draft, Supervision, Funding acquisition, Formal analysis.

## Declaration of competing interest

The authors declare that they have no known competing financial interests or personal relationships that could have appeared to influence the work reported in this paper.
