# Supplementary material for: Accelerated polyethylene biodegradation in soil through carbon nanomaterial-primed rhizospheres
Source: Environ Sci Ecotechnol. 2026 Sep 23;34:100772. doi: 10.1016/j.ese.2026.100772 (PMC13634778; doi:10.1016/j.ese.2026.100772)
Supplement: Supplementary file 1 [file mmc1.docx]

**SUPPLEMENTARY INFORMATION I**

Haoran Liu ^1^*, Lena Ciric ^1^, Manpreet Bhatti ^1^

^1^ UCL Department of Civil, Environmental and Geomatic Engineering, London, WC1E 6BT, UK

*This is the corresponding author

# Summary Information

Number of pages: 12

Figures: S1-S6

Tables: S1-S3

**Table of** **Contents**

**[Section I. Nanomaterial(NM) characterization	1](#_Toc12190)**

[Table S1. Physicochemical characterization of MWCNTs and GO	2](#_Toc20306)

[Figure S1. Characterization of Two NMs, including SEM and AFM	3](#_Toc13935)

**[Section II. Plant growth and plastic degradation	3](#_Toc6423)**

[Figure S2. Pre-experiment results and photographs of the plant experiments	4](#_Toc10514)

[Figure S3. Plant growth indicators across all NM added groups from 0 to 90d	5](#_Toc13738)

[Figure S4.FT-IR and XPS of plastic films	6](#_Toc24579)

**[Section III Microbial Communities	6](#_Toc20971)**

[Figure S5. Shannon and Simpson diversity indices of soil microbial communities across all treatment groups at 30 and 90 days.	7](#_Toc31692)

[Figure S6. Venn diagram.	7](#_Toc18442)

[Table S2. Listed Genus-level microorganisms and degradation functions or sensitivity to plastics	8](#_Toc27552)

[Table S3. KEGG Orthology (KO) Information Table	9](#_Toc30244)

**[Section IV. Expanded risk‑benefit, cost, and safety assessment	10](#_Toc3123)**

**[References	11](#_Toc10466)**

Note: The Table of Contents provides concise titles for quick navigation.

# Section I. Nanomaterial(NM) characterization

The detailed physicochemical properties of both NMs, including size and purity, are summarized from the instruction manual provided by the supplier (Brand: Shilpent) and presented in Table S1. The following methods were employed to test the other indicators or to verify the indicators stated in the instruction manual, including:

(1) Scanning electron microscopy (SEM): Zeiss SEM (Germany), operated at an accelerating voltage of 5 kV.
(2) Atomic force microscopy (AFM): Bruker Dimension Icon (USA), tapping mode.
(3) Brunauer-Emmett-Teller (BET) analysis: Micromeritics ASAP 2460 (USA), nitrogen adsorption at 77 K.
(4) Fourier-transform infrared spectroscopy (FT-IR): Bruker VERTEX 80v (Germany), spectra recorded in the range of 4000-400 cm^-1^.
(5) X-ray photoelectron spectroscopy (XPS): Thermo Fisher Scientific ESCALAB Xi^+^ (UK), monochromatic Al Kα radiation (1486.6 eV).
(6) Zeta potential: Brookhaven 90Plus PALS (Nanobrook) nanoparticle size and zeta potential analyzer.

### Table S1. Physicochemical characterization of MWCNTs and GO

| Parameter | MWCNTs | GO |
| --- | --- | --- |
| Purity (carbon basis; instruction manual) | >99.98 wt% | >99 wt% |
| Outer Diameter (instruction manual) | 5–100 nm | 0.8–2 nm |
| Lateral size (instruction manual) | 1-100 μm | 5–10 μm |
| Specific surface area (BET-instruction manual) | 100-400 m^2^ g^-1^ | 110-250  m^2^ g^-1^ |
| Specific surface area (BET-tested) | 89.77 ± 3.22 m^2^ g^-1^ | 63.60 ± 4.50  m^2^ g^-1^ |
| Zeta potential (pH 7, H_2_O, tested) | −25.6 ± 2.1mV | −32.8  ± 1.9mV |
| Residual metal catalyst (instruction manual) | Fe <0.5 wt% | - |

It is worth noting that the BET standard value of MWCNTs and GO provided in the manual is different from the measured BET value. This indicates that MWCNTs and GO may have aggregated during actual use. The same difference is observed in the comparison between the SEM image of GO and the standard size. It is clearly seen that the GO in the SEM image is larger and might also be a cluster.

### Figure S1. Characterization of Two NMs, including: (a) SEM of MWCNTs; (b) SEM of GO; (c) XPS of GO; (d) XPS of MWCNTs; (e) AFM of MWCNTs; (f) AFM of GO; (g) FTIR of both MWCNTs and GO.

# Section II. Plant growth and plastic degradation

### Figure S2. Pre-experiment results and photographs of the plant experiments, including (a) degradation rate of PE film in the 45-day hydroponic pre-experiment with 100 mg kg^-1^ NMs; (b) photographs of germination tests;

### (c) Photos of pots before thinning and insect-trapping boards, PE+CNTs200 (left) and PE only control group (right);

### (d) photographs of the greenhouse pot experiment and nodules observed after destructive sampling at 60th d (inset at lower left).

In the 45-day hydroponic pre-experiment, 100 mg kg^-1^ NMs promoted PE phytoremediation (Figure S2a). The pot status before and after thinning is shown in Figures S2c and S2d, respectively. The comparison highlights the importance of this procedure, as excessively dense plants interfere with growth and data collection. Notably, the insect trap from the MWCNTs200-amended group contained visibly fewer flying insects than that from the PE-only control group (Figure S2c).

### Figure S3. Plant growth indicators across all NM added groups from 0 to 90d. Data are grouped by treatment, including PE+Plants+MWCNTs and PE+Plants+GO at concentrations of 100, 150, and 200 mg kg^-1^. Indicators include shoot height (cm), root length (cm), fresh weight (g), and number of flying insects (individuals). Note: Data for the 150 mg kg^-1^ treatment were obtained from a separate experimental batch. See Methods for details.

### Figure S4.(a) FT-IR spectra of PE films comparing plant-NM combined treatments with abiotic and blank controls. (b) XPS elemental analysis of corresponding PE film surfaces. (c) Examples of XPS sample images. Note：Due to the low readability of the original images obtained from the XPS sample measurement, the peak areas were directly used for plotting in Figures 3 and Figure S4. The original images of any XPS sample determination can be obtained by contacting the author.

FT-IR spectra of PE films from different treatments are presented in Figure S4a, including plant-NM combined groups (PE+Plants+MWCNTs, PE+Plants+GO), abiotic NM controls (PE+MWCNTs, PE+GO), plant-only (PE+Plants), PE-only, and Rare PE. The PE+Plants+MWCNTs and PE+Plants+GO samples showed the highest absorbance at the characteristic PE peaks, followed by the PE+Plants group. In contrast, the abiotic NM-only controls (PE+MWCNTs, PE+GO) and the PE-only control showed only slightly higher absorbance than the unaged Rare PE.

The corresponding XPS analysis (Figure S4b) confirmed these trends. The plant-NM combined treatments exhibited the highest O1s peak areas, while the abiotic NM-only and PE-only controls showed only minor increases in O1s signal compared to Rare PE. N1s signals were detectable in all plant-containing treatments, whereas S2p signals remained negligible.

# Section III Microbial Communities

In contrast to the Chao1 richness estimator, the Shannon and Simpson indices displayed consistently high values across all treatment groups, with relatively minor absolute differences (Figure S5). This pattern indicates a high level of microbial diversity and evenness, suggesting that the communities maintained considerable functional redundancy or stability irrespective of treatment. Statistical analysis revealed that most experimental groups differed significantly from the control (PE only), with the most pronounced differences observed in the bottom layer at 90 days. Furthermore, within the same treatment, the Simpson index showed significant differences (p < 0.05) between the bottom and surface layers specifically in the two groups exhibiting the highest PE degradation rates, namely MWCNTs200 and GO150.

### Figure S5. Shannon and Simpson diversity indices of soil microbial communities across all treatment groups at 30 and 90 days. Data are presented for both surface and bottom layers. Significant differences between treatments and the PE-only control are indicated by asterisks (p < 0.05, **p < 0.01, **p < 0.001; one-way ANOVA). Note: Batch details as in Figure S3.

Venn diagrams were used to illustrate the shared and unique microbial taxa among different treatment groups (Allain et al., 2024, Feng et al., 2020). As shown in Figure S6a, the MWCNTs‑amended groups shared 686 taxa between the surface and bottom layers at both 30 and 90 days, which was substantially higher than the 191 shared taxa between the MWCNTs‑amended groups and the two control groups at 90 days. This pattern directly demonstrates that MWCNTs had already shaped a distinct microbial community by 30 days, and this community persisted through 90 days, remaining clearly differentiated from the controls.

### Figure S6. Venn diagram, including (a)PE only 90d vs PE+Plants+MWCNTs200 Surface 90d vs PE+Plants+MWCNTs200 Bottom 90d vs PE+plants only surface 90d; (b) PE+Plants+MWCNTs200 Surface 90d vs PE+Plants+MWCNTs200 Bottom 90d vs PE+Plants+MWCNTs200 Surface 30d vs PE+Plants+MWCNTs200 Bottom 30d.

The reported roles of key genera detected in this study are summarised in Table S2, covering plastic degradation, other pollutant degradation, and plastic‑sensitive taxa. The putative functions of differentially enriched KEGG Orthology identifiers, including transporters, stress response regulators, a detoxification enzyme, and a fatty acid biosynthesis enzyme, are detailed in Table S3. These two tables provide a functional framework that links community composition to the proposed PE degradation pathway involving C-C bond cleavage, detoxification, and transmembrane transport.

### Table S2. Listed Genus-level microorganisms and degradation functions or sensitivity to plastics

| Name | Target/Sources of coercion | Shape/status | Media/Machine | Materials or Plants | Function/Reason | References |
| --- | --- | --- | --- | --- | --- | --- |
| Plastic degradation | | | | | | |
| *Rhodanobacter* | PE | Film | Soil | - | Plastic degradation | (Wang et al., 2023) |
| *Pseudolabrys* | PBAT^1^ | Film | Soil | - | The soluble sugar and nitrate concentration of plants | (Gao et al., 2025) |
| *Opitutus* | PE and PBAT | Film | Soil | Maize | N cycling increased | (Zhang et al., 2024) |
| *Gemmatimonas* | HDPE, PLA and PHBV^1^ | Pellet | Freshwater | - | Plastic degradation | (Nguyen et al., 2023) |
| *Novosphingobium* | PS | NPs^1^ | Wastewater | - | Plastic resistance and degradation | (Liu et al., 2025b) |
|  | PS | Foam | Epilittoral zone | Mangroves | Plastic degradation | (Liu et al., 2023) |
| Degradation of other organic pollutants | | | | | | |
| *Oleiharenicola* | Methane | Gas | Methane-Fed Bioreactor | - | Methane-derived carbon cycling | (Danilova et al., 2025) |
| *Steroidobacter* | BaP^1^ | - | Soil | Rice straw amendment | Degradation | (Zhang et al., 2025) |
|  | PCBs^1^ | - | E-waste-contaminated soil | - | Degradation | (Wang et al., 2022) |
| *Flavobacterium* | BaP | - | Epilittoral zone | Mangroves | Degradation | (Meng et al., 2024) |
| *Mucilaginibacter* | Phenol | - | Wastewater | SWCNTs^1^ | Degradation by EPS | (Qu et al., 2015) |
|  | Cellulose | - | Agar plates | - | Degradation by EPS | (Wang et al., 2020) |
| Reduced by plastic (Plastic sensitivity) | | | | | | |
| *Gemmatimonas* | HDPE, PS and PP^1^ | MPs | Soil | - | C/N reduced | (Qin et al., 2023) |
| *Acidothermus* | PE | MPs | Soil | - | C/N reduced | (Li et al., 2024) |
| *Dongia* | Al-coated PET^1^ | Film | Soil | - | C reduced | (Wang et al., 2025) |

Note: 1. PBAT is Poly (butylene adipate-co-terephthalate); HDPE is High-Density Polyethylene; PLA is Polylactic Acid; PHBV is Poly(3-hydroxybutyrate-co-3-hydroxyvalerate); BaP is Benzo[a]pyrene; PCBs is Polychlorinated Biphenyls; PP is Polypropylene; PET is Polyethylene Terephthalate; SWCNTs is Single-Walled Carbon Nanotubes; NPs is nanoplastics.

2. Microorganisms from Figure 4-5 not listed in the table were not reported under the three conditions.

### Table S3. KEGG Orthology (KO) Information Table

| KO ID | Pathway Name | Core Functional Description | Official KEGG Link |
| --- | --- | --- | --- |
| K07090 | membrane protein, putative transporter | Putative membrane transporter protein, involved in basic transmembrane transport of substances, with function not yet fully characterized | [https://www.genome.jp/dbget-bin/www_bget?ko:K07090](https://link.wtturl.cn/?target=https://www.genome.jp/dbget-bin/www_bget?ko:K07090&scene=im&aid=497858&lang=zh) |
| K03704 | glutathione-regulated potassium-efflux system protein | Glutathione-regulated potassium efflux system protein, participates in bacterial osmotic pressure regulation and oxidative stress response | [https://www.genome.jp/dbget-bin/www_bget?ko:K03704](https://link.wtturl.cn/?target=https://www.genome.jp/dbget-bin/www_bget?ko:K03704&scene=im&aid=497858&lang=zh) |
| K03406 | two-component system, OmpR family, sensor histidine kinase | Sensor histidine kinase of the OmpR family two-component system, responsible for sensing environmental signals and regulating downstream gene expression | [https://www.genome.jp/dbget-bin/www_bget?ko:K03406](https://link.wtturl.cn/?target=https://www.genome.jp/dbget-bin/www_bget?ko:K03406&scene=im&aid=497858&lang=zh) |
| K02040 | putative iron-siderophore transport system permease protein | Putative permease protein of the iron-siderophore transport system, involved in bacterial iron uptake process | [https://www.genome.jp/dbget-bin/www_bget?ko:K02040](https://link.wtturl.cn/?target=https://www.genome.jp/dbget-bin/www_bget?ko:K02040&scene=im&aid=497858&lang=zh) |
| K02003 | putative ABC transport system ATP-binding protein | Putative ATP-binding protein of the ABC transport system, provides energy for transmembrane transport of various substrates via ATP hydrolysis | [https://www.genome.jp/dbget-bin/www_bget?ko:K02003](https://link.wtturl.cn/?target=https://www.genome.jp/dbget-bin/www_bget?ko:K02003&scene=im&aid=497858&lang=zh) |
| K01999 | branched-chain amino acid transport system ATP-binding protein | ATP-binding protein of the branched-chain amino acid transport system, participates in active transport of leucine, isoleucine and valine | [https://www.genome.jp/dbget-bin/www_bget?ko:K01999](https://link.wtturl.cn/?target=https://www.genome.jp/dbget-bin/www_bget?ko:K01999&scene=im&aid=497858&lang=zh) |
| K01997 | polar amino acid transport system ATP-binding protein | ATP-binding protein of the polar amino acid transport system, participates in transmembrane transport of polar amino acids (e.g., serine, threonine) | [https://www.genome.jp/dbget-bin/www_bget?ko:K01997](https://link.wtturl.cn/?target=https://www.genome.jp/dbget-bin/www_bget?ko:K01997&scene=im&aid=497858&lang=zh) |
| K01996 | branched-chain amino acid transport system permease protein | Permease protein of the branched-chain amino acid transport system, constitutes the intramembrane domain of the transport channel | [https://www.genome.jp/dbget-bin/www_bget?ko:K01996](https://link.wtturl.cn/?target=https://www.genome.jp/dbget-bin/www_bget?ko:K01996&scene=im&aid=497858&lang=zh) |
| K00799 | glutathione S-transferase | Glutathione S-transferase (GST), catalyzes the conjugation of glutathione with exogenous/endogenous electrophilic compounds, mediates detoxification and antioxidant processes | [https://www.genome.jp/dbget-bin/www_bget?ko:K00799](https://link.wtturl.cn/?target=https://www.genome.jp/dbget-bin/www_bget?ko:K00799&scene=im&aid=497858&lang=zh) |
| K00059 | 3-oxoacyl-[acyl-carrier protein] reductase | 3-oxoacyl-[acyl-carrier protein] reductase (FabG), participates in fatty acid biosynthesis, catalyzes the reduction reaction of β-ketoacyl groups | [https://www.genome.jp/dbget-bin/www_bget?ko:K00059](https://link.wtturl.cn/?target=https://www.genome.jp/dbget-bin/www_bget?ko:K00059&scene=im&aid=497858&lang=zh) |

# Section IV. Expanded risk‑benefit, cost, and safety assessment

**4.1 Risk‑benefit balance: NMs vs. plastic pollution**

The long‑term fate of MWCNTs and GO in soil (e.g., persistence, potential for aggregation, and impact on soil biota) requires further investigation. However, it must be weighed against the alternative, where leaving polyethylene to fragment into microplastics (MPs) and nanoplastics (NPs) over decades, will inevitably generate persistent, bioavailable plastic debris that is already known to cause ecotoxicity and enter food chains based on our previous study (Liu et al., 2025a). The NM concentrations used here (100-200 mg kg^-1^) are within the typical testing range for phytoremediation and are not intended as an open‑release scenario. A life‑cycle perspective suggests that the net environmental benefit of accelerated PE degradation may outweigh the localized NM footprint, provided that appropriate risk management measures are implemented.

**4.2 Worker safety and regulatory acceptance**

MWCNTs share certain physical similarities with asbestos (e.g., high aspect ratio, biopersistence), which raises legitimate worker safety concerns. However, the actual health risk strongly depends on the form of exposure. In our proposed application, NMs are introduced as a dilute soil amendment (≤200 mg kg^-1^) and are not aerosolized, which substantially mitigates inhalation risk. Standard engineering controls (e.g., dust suppression, personal protective equipment) would be required for large‑scale handling, as routinely applied for fine powders. Therefore, while regulatory acceptance will require careful evaluation, the risks are manageable and distinct from occupational exposure in production facilities.

**4.3 Cost feasibility and future scalability**

At current market prices, MWCNTs and GO are more expensive than conventional soil amendments, which could limit immediate large‑scale adoption. Nevertheless, industrial‑scale production of carbon NMs is expanding rapidly. For example, a thousand‑ton‑per‑year production line for CNTs from natural gas began operation in China in early 2026 (ChemNet, 2026), and manufacturers such as NoPo are achieving significant cost reductions through in‑house reactor design and local sourcing (Verma, 2026). As production scales up, the unit cost of both MWCNTs and GO is expected to decrease substantially. For the concentration used here (200 mg kg⁻¹), the material cost per hectare is estimated to be on the order of a few hundred US dollars, which is within the range of other remediation technologies when long‑term benefits are considered. Thus, economic feasibility is likely to improve in the near future.

# References

ALLAIN, A., ALEXIS, M. A., BRIDOUX, M. C., SHIROKOVA, L. S., PAYANDI-ROLLAND, D., POKROVSKY, O. S. & ROUELLE, M. 2024. The specific molecular signature of dissolved organic matter extracted from different arctic plant species persists after biodegradation. *Soil Biology and Biochemistry,* 193.

CHEMNET. 2026. China's first thousand-ton-scale production line for carbon nanotubes from natural gas begins operation. *ChemNet*.

DANILOVA, O. V., SALOVA, V. D., OSHKIN, I. Y., NAUMOFF, D. G., IVANOVA, A. A., SUZINA, N. E. & DEDYSH, S. N. 2025. Isolation of Ultra-Small Opitutaceae-Affiliated Verrucomicrobia from a Methane-Fed Bioreactor. *Microorganisms,* 13.

FENG, L. J., SUN, X. D., ZHU, F. P., FENG, Y., DUAN, J. L., XIAO, F., LI, X. Y., SHI, Y., WANG, Q., SUN, J. W., LIU, X. Y., LIU, J. Q., ZHOU, L. L., WANG, S. G., DING, Z., TIAN, H., GALLOWAY, T. S. & YUAN, X. Z. 2020. Nanoplastics Promote Microcystin Synthesis and Release from Cyanobacterial Microcystis aeruginosa. *Environ Sci Technol,* 54**,** 3386-3394.

GAO, W., TU, Z., YIN, X., MING, S. & CAI, K. 2025. Effects of PBAT biodegradable mulch on lettuce (Lactuca sativa L.) physiology and soil microbial community: Based on a long-term degradation trial. *Ecotoxicol Environ Saf,* 302**,** 118734.

LI, S., ZHONG, L., ZHANG, B., FAN, C., GAO, Y., WANG, M., XIAO, H. & TANG, X. 2024. Microplastics induced the differential responses of microbial-driven soil carbon and nitrogen cycles under warming. *J Hazard Mater,* 465**,** 133141.

LIU, H., CIRIC, L. & BHATTI, M. 2025a. Effects of nanoplastics and compound pollutants containing nanoplastics on plants, microorganisms and rhizosphere systems: A review. *Ecotoxicol Environ Saf,* 294**,** 118084.

LIU, R., ZHAO, S., ZHANG, B., LI, G., FU, X., YAN, P. & SHAO, Z. 2023. Biodegradation of polystyrene (PS) by marine bacteria in mangrove ecosystem. *J Hazard Mater,* 442**,** 130056.

LIU, X., YANG, Y., GRAHAM, N. J. D., TAKIZAWA, S. & NG, H. Y. 2025b. Deciphering membrane biofouling induced by micro-/nano-plastics in nanofiltration: Metagenomic insights and spacer-driven mitigations. *Water Res,* 281**,** 123682.

MENG, S., PENG, T., LIU, Y., ZHANG, S., QIAN, Z., HUANG, T., XIE, Q., GU, J. D. & HU, Z. 2024. Novel insights into the synergetic degradation of pyrene by microbial communities from mangroves in China. *J Hazard Mater,* 469**,** 133907.

NGUYEN, N. H. A., MARLITA, M., EL-TEMSAH, Y. S., HRABAK, P., RIHA, J. & SEVCU, A. 2023. Early stage biofilm formation on bio-based microplastics in a freshwater reservoir. *Sci Total Environ,* 858**,** 159569.

QIN, P., LI, T., CUI, Z., ZHANG, H., HU, X., WEI, G. & CHEN, C. 2023. Responses of bacterial communities to microplastics: More sensitive in less fertile soils. *Sci Total Environ,* 857**,** 159440.

QU, Y., MA, Q., DENG, J., SHEN, W., ZHANG, X., HE, Z., VAN NOSTRAND, J. D., ZHOU, J. & ZHOU, J. 2015. Responses of microbial communities to single-walled carbon nanotubes in phenol wastewater treatment systems. *Environ Sci Technol,* 49**,** 4627-35.

VERMA, R. 2026. Powering Tomorrow with Scalable SWCNT Innovation. *Bisinfotech*.

WANG, K., FLURY, M., KUZYAKOV, Y., ZHANG, H., ZHU, W. & JIANG, R. 2025. Aluminum and microplastic release from reflective agricultural films disrupt microbial communities and functions in soil. *J Hazard Mater,* 491**,** 137891.

WANG, P., LIU, J., HAN, S., WANG, Y., DUAN, Y., LIU, T., HOU, L., ZHANG, Z., LI, L. & LIN, Y. 2023. Polyethylene mulching film degrading bacteria within the plastisphere: Co-culture of plastic degrading strains screened by bacterial community succession. *J Hazard Mater,* 442**,** 130045.

WANG, S., LI, J., JIANG, L., WANG, S., ZHAO, X., DAI, Y., LUO, C. & ZHANG, G. 2022. The influence of anaerobic dechlorination on the aerobic degradation of PCBs in e-waste-contaminated soils in an anaerobic-aerobic two-stage treatment. *Sci Total Environ,* 844**,** 157195.

WANG, Z. Y., WANG, R. X., ZHOU, J. S., CHENG, J. F. & LI, Y. H. 2020. An assessment of the genomics, comparative genomics and cellulose degradation potential of Mucilaginibacter polytrichastri strain RG4-7. *Bioresour Technol,* 297**,** 122389.

ZHANG, D., WAN, L. C., WANG, M. X. & ZHOU, Z. F. 2025. Bio-stimulating effect and mechanism of rice straw amendment for enhancing the elimination of soil benzo[a]pyrene. *Ecotoxicol Environ Saf,* 302**,** 118570.

ZHANG, H., SHU, D., ZHANG, J., LIU, X., WANG, K. & JIANG, R. 2024. Biodegradable film mulching increases soil microbial network complexity and decreases nitrogen-cycling gene abundance. *Sci Total Environ,* 933**,** 172874.
